# Retracted: Proanthocyanidins Antagonize Arsenic-Induced Oxidative Damage and Promote Arsenic Methylation through Activation of the Nrf2 Signaling Pathway

**DOI:** 10.1155/2021/3547620

**Published:** 2021-01-22

**Authors:** Oxidative Medicine and Cellular Longevity

Oxidative Medicine and Cellular Longevity has retracted the article titled “Proanthocyanidins Antagonize Arsenic-Induced Oxidative Damage and Promote Arsenic Methylation through Activation of the Nrf2 Signaling Pathway” [1]. As raised on PubPeer [2], the article was found to contain images with signs of duplication in Figure 2, specifically the lower left of the flow cytometry shown in panels 2(a)1, 2(a)2, 2(b)3, and 2(b)4.

We asked the authors to provide the original uncropped and unadjusted images used to create all the figures, and all the raw data, i.e. all the underlying measurements for the statistics and averages shown in the graphs and tables. The authors did not provide the original images and raw data. However, they provided a corrected figure, which is available as Supplementary Materials. We consulted our Editorial Board, who recommended retraction.

## Supplementary Materials

Supplementary Materials Figure duplication in Figure 2 of OMCL/8549035. (Supplementary Materials.docx). Corrected figure files (Supplementary Materials.rar).
