# Supplementary material for: Retracted: Proanthocyanidins Antagonize Arsenic-Induced Oxidative Damage and Promote Arsenic Methylation through Activation of the Nrf2 Signaling Pathway
Source: Oxid Med Cell Longev. 2021 Jan 22;2021:3547620. doi: 10.1155/2021/3547620 (PMC7846395; doi:10.1155/2021/3547620)
Supplement: Supplementary Materials — Figure duplication in Figure 2 of OMCL/8549035. (Supplementary Materials.docx). Corrected figure files (Supplementary Materials.rar). [file 3547620.f1.zip › 3547620.f1/raw data/3.apoptosis/apoptosis(figure2).pdf]

11-15-2 报告

标本名：11-15-2　　　　检验时间：2017/11/15 18:27

仪器：FACSAriaII　　　　软件：NovoExpress 1.3.1

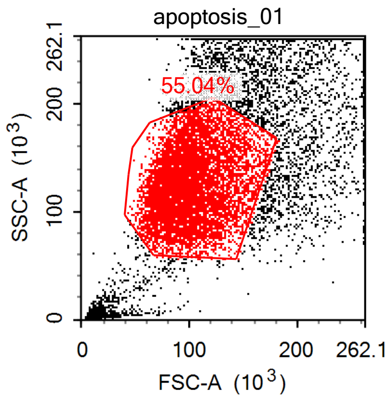

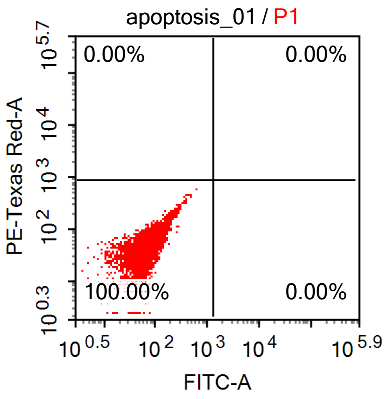

样本统计表格 - apoptosis_01

Gate	Count	% Parent	X	Y	Median X	Median Y
All	14,864					
P1	8,181	55.04%	FSC-A	SSC-A	98,235	123,697
Q2-1	0	0.00%	FITC-A	PE-Texas Red-A	0	0
Q2-2	0	0.00%	FITC-A	PE-Texas Red-A	0	0
Q2-3	8,181	100.00%	FITC-A	PE-Texas Red-A	64	51
Q2-4	0	0.00%	FITC-A	PE-Texas Red-A	0	0

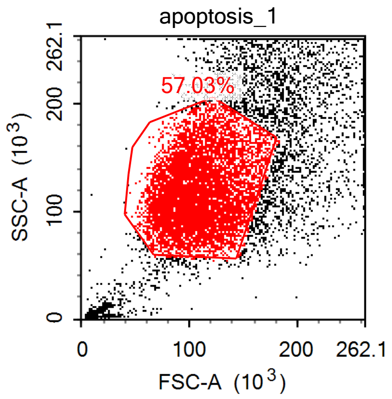

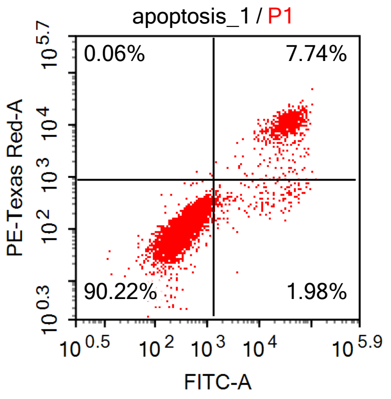

样本统计表格 - apoptosis_1

Gate	Count	% Parent	X	Y	Median X	Median Y
All	13,573					
P1	7,741	57.03%	FSC-A	SSC-A	105,050	115,889
Q2-1	5	0.06%	FITC-A	PE-Texas Red-A	1,076	1,173
Q2-2	599	7.74%	FITC-A	PE-Texas Red-A	35,534	10,512
Q2-3	6,984	90.22%	FITC-A	PE-Texas Red-A	332	109
Q2-4	153	1.98%	FITC-A	PE-Texas Red-A	10,654	386

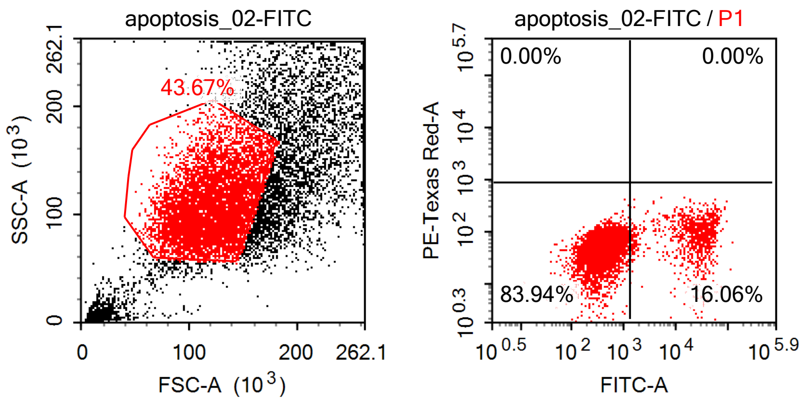

样本统计表格 - apoptosis_02-FITC

Gate	Count	% Parent	X	Y	Median X	Median Y
All	15,239					
P1	6,655	43.67%	FSC-A	SSC-A	120,401	106,460
Q2-1	0	0.00%	FITC-A	PE-Texas Red-A	0	0
Q2-2	0	0.00%	FITC-A	PE-Texas Red-A	0	0
Q2-3	5,586	83.94%	FITC-A	PE-Texas Red-A	363	48
Q2-4	1,069	16.06%	FITC-A	PE-Texas Red-A	26,987	80

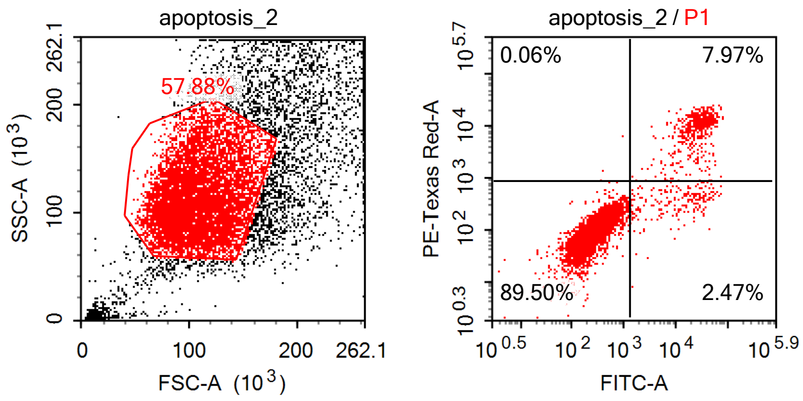

样本统计表格 - apoptosis_2

Gate	Count	% Parent	X	Y	Median X	Median Y
All	13,311					
P1	7,705	57.88%	FSC-A	SSC-A	107,284	111,392
Q2-1	5	0.06%	FITC-A	PE-Texas Red-A	478	923
Q2-2	614	7.97%	FITC-A	PE-Texas Red-A	28,569	11,097
Q2-3	6,896	89.50%	FITC-A	PE-Texas Red-A	251	98
Q2-4	190	2.47%	FITC-A	PE-Texas Red-A	13,598	379

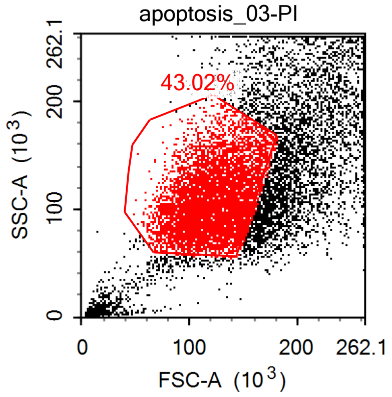

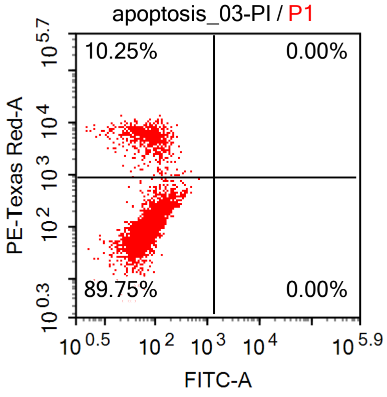

样本统计表格 - apoptosis_03-PI

Gate	Count	% Parent	X	Y	Median X	Median Y
All	14,828					
P1	6,379	43.02%	FSC-A	SSC-A	121,655	104,764
Q2-1	654	10.25%	FITC-A	PE-Texas Red-A	74	5,819
Q2-2	0	0.00%	FITC-A	PE-Texas Red-A	0	0
Q2-3	5,725	89.75%	FITC-A	PE-Texas Red-A	76	103
Q2-4	0	0.00%	FITC-A	PE-Texas Red-A	0	0

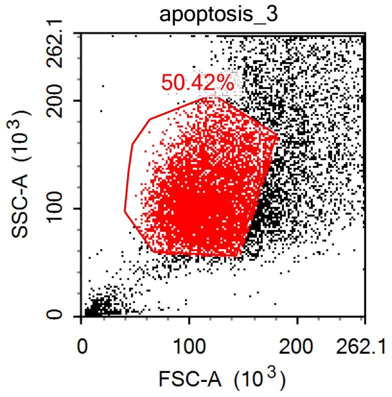

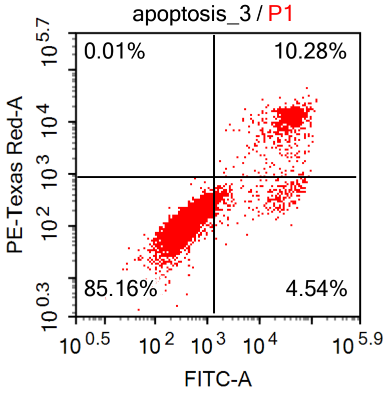

样本统计表格 - apoptosis_3

Gate	Count	% Parent	X	Y	Median X	Median Y
All	14,023					
P1	7,071	50.42%	FSC-A	SSC-A	114,919	108,459
Q2-1	1	0.01%	FITC-A	PE-Texas Red-A	550	823
Q2-2	727	10.28%	FITC-A	PE-Texas Red-A	37,904	12,193
Q2-3	6,022	85.16%	FITC-A	PE-Texas Red-A	375	123
Q2-4	321	4.54%	FITC-A	PE-Texas Red-A	12,934	375

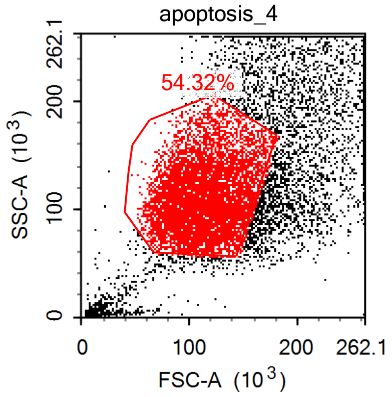

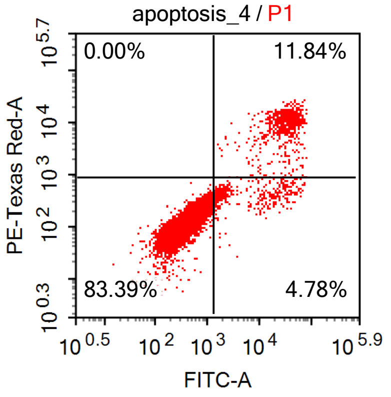

样本统计表格 - apoptosis_4

Gate	Count	% Parent	X	Y	Median X	Median Y
All	13,531					
P1	7,350	54.32%	FSC-A	SSC-A	113,391	107,958
Q2-1	0	0.00%	FITC-A	PE-Texas Red-A	0	0
Q2-2	870	11.84%	FITC-A	PE-Texas Red-A	33,177	11,684
Q2-3	6,129	83.39%	FITC-A	PE-Texas Red-A	379	127
Q2-4	351	4.78%	FITC-A	PE-Texas Red-A	7,863	422

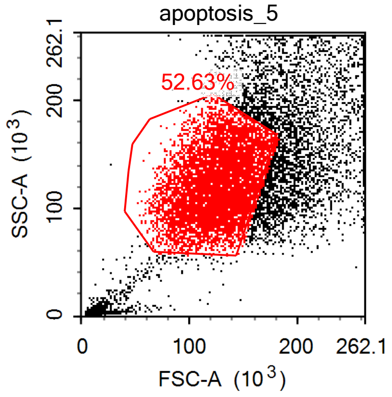

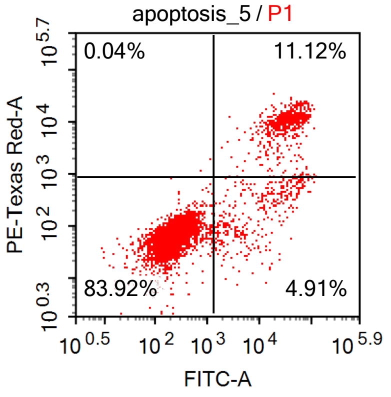

样本统计表格 - apoptosis_5

Gate	Count	% Parent	X	Y	Median X	Median Y
All	12,918					
P1	6,799	52.63%	FSC-A	SSC-A	125,030	121,129
Q2-1	3	0.04%	FITC-A	PE-Texas Red-A	713	1,182
Q2-2	756	11.12%	FITC-A	PE-Texas Red-A	38,260	11,249
Q2-3	5,706	83.92%	FITC-A	PE-Texas Red-A	205	69
Q2-4	334	4.91%	FITC-A	PE-Texas Red-A	10,014	187

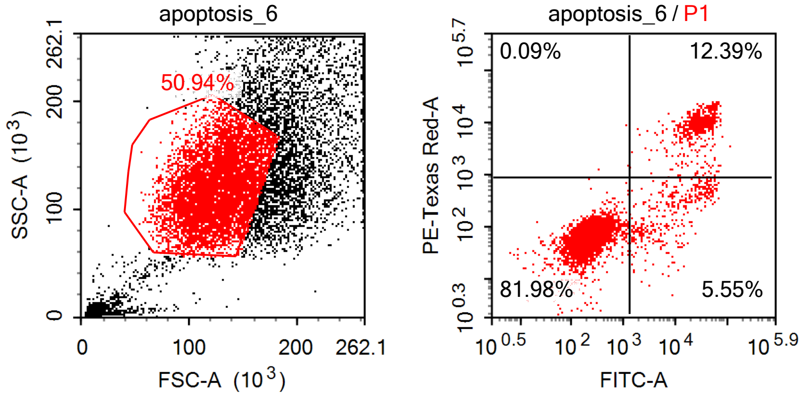

样本统计表格 - apoptosis_6

Gate	Count	% Parent	X	Y	Median X	Median Y
All	13,169					
P1	6,708	50.94%	FSC-A	SSC-A	126,571	120,491
Q2-1	6	0.09%	FITC-A	PE-Texas Red-A	1,097	1,471
Q2-2	831	12.39%	FITC-A	PE-Texas Red-A	31,615	10,519
Q2-3	5,499	81.98%	FITC-A	PE-Texas Red-A	198	66
Q2-4	372	5.55%	FITC-A	PE-Texas Red-A	9,329	251

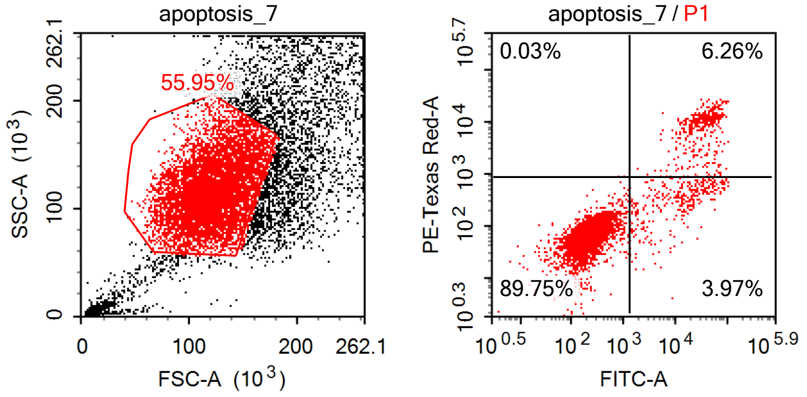

样本统计表格 - apoptosis_7

Gate	Count	% Parent	X	Y	Median X	Median Y
All	13,025					
P1	7,288	55.95%	FSC-A	SSC-A	121,776	115,545
Q2-1	2	0.03%	FITC-A	PE-Texas Red-A	884	911
Q2-2	456	6.26%	FITC-A	PE-Texas Red-A	38,540	10,742
Q2-3	6,541	89.75%	FITC-A	PE-Texas Red-A	203	67
Q2-4	289	3.97%	FITC-A	PE-Texas Red-A	14,132	355

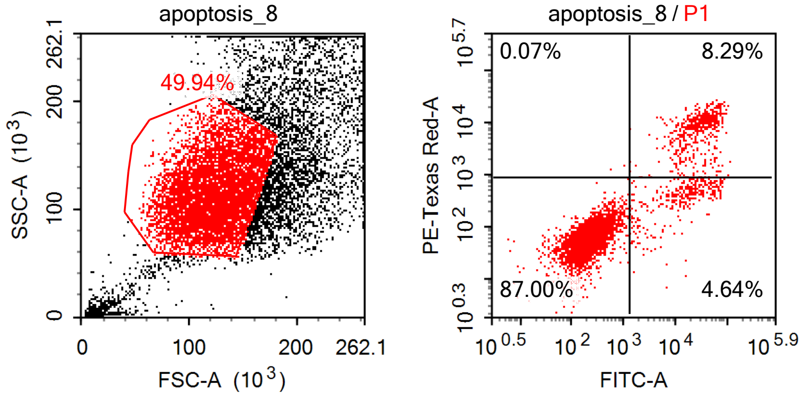

样本统计表格 - apoptosis_8

Gate	Count	% Parent	X	Y	Median X	Median Y
All	13,474					
P1	6,729	49.94%	FSC-A	SSC-A	123,525	114,581
Q2-1	5	0.07%	FITC-A	PE-Texas Red-A	1,245	1,595
Q2-2	558	8.29%	FITC-A	PE-Texas Red-A	32,763	9,984
Q2-3	5,854	87.00%	FITC-A	PE-Texas Red-A	203	68
Q2-4	312	4.64%	FITC-A	PE-Texas Red-A	14,538	417
