# Supplementary material for: Retracted: Proanthocyanidins Antagonize Arsenic-Induced Oxidative Damage and Promote Arsenic Methylation through Activation of the Nrf2 Signaling Pathway
Source: Oxid Med Cell Longev. 2021 Jan 22;2021:3547620. doi: 10.1155/2021/3547620 (PMC7846395; doi:10.1155/2021/3547620)
Supplement: Supplementary Materials — Figure duplication in Figure 2 of OMCL/8549035. (Supplementary Materials.docx). Corrected figure files (Supplementary Materials.rar). [file 3547620.f1.zip › 3547620.f1/raw data/8.The review process/8549035-final manuscript.pdf]

Proanthocyanidins antagonises arsenic-induced oxidative damage and promotes arsenic methylation through activation of Nrf2 signalling pathway

Mengchuan Xu[*], Qiang Niu[*], Yunhua Hu, Gangling Feng, Haixia Wang, Shugang Li[#]

Department of Public Health, Medical College, Shi Hezi University (832000), Xin Jiang, China

[*] Co-first authors: Mengchuan Xu and Qiang Niu contributed equally to this work.

[#] Corresponding Author: Shugang Li[#], Department of Public Health, Shihezi University School of Medicine, Shihezi, Xinjiang, 832000 (E-mail: lishugang@ymail.com).

E-mail address: xmc2015202205@163.com (M. X.); shzniuqiang@sina.com (Q. N.); hyh6133@sina.com (Y. H.); fgl_shzu@163.com (G. F.); wanghaixiawza@163.com (H. W.); lishugang@ymail.com (S. L.).

Abstract

Purpose To investigate the effects of grape seed proanthocyanidins extract (GSPE) on oxidative damage and arsenic (As) methylation, and to clarify the role of Nrf2 in the process.

Methods L-02 cells were treated with arsenic (25μM) and GSPE (10, 25, 50mg/L) for 24 h. Cell viability was analysed by MTT assay. Cell apoptosis and ROS fluorescence were detected by flow cytometry. Oxidative stress marker levels were measured using commercial kits. mRNA and protein expression were detected by qRT-PCR and western blotting. The cellular concentrations of methylation products were measured by HPLC-HGAFS. Arsenic methylation ability of cells was determined.

Results Cell survival rate was significantly lower in As group than in control group ($P < 0.05$), while cell apoptosis increased and the number of apoptotic cells decreased gradually after GSPE intervention. Superoxide dismutase, glutathione, and sulfhydryl levels in intervention group were significantly higher ($P < 0.05$), while MDA and ROS levels were significantly lower ($P < 0.05$) than those in As group. The mRNA and protein expression of Nrf2, HO-1, NQO1, and glutathione-S-transferase increased in As + GSPE group compared with that in As group ($P < 0.05$). GSPE significantly increased methylated As level, primary methylation index, secondary methylation index, average growth rate of methylation, and average methylation speed compared with GSPE untreated group ($P < 0.05$). After Nrf2 inhibition, the effect of GSPE decreased significantly.

Conclusion GSPE activates Nrf2 signalling pathway to antagonise As-induced oxidative damage and to promote As methylation metabolism. Therefore, GSPE may be a potential agent for relieving As-induced hepatotoxicity.

Keywords: grape seed proanthocyanidins extract; arsenic; antioxidant; arsenic methylation, Nrf2 signalling pathway

1. Introduction

Arsenic is a metalloid toxin and carcinogen present widely in soil, rocks, and water[1]. Our previous studies have proved that arsenic can caused reproductive toxicity[2, 3], but the mechanism of arsenic toxicity is not completely clear and should be further studied. In recent years, China has gradually become one of the countries with the highest impact and incidence rate of endemic arsenism[4].

The liver is one of the important target organs of arsenic toxicity. Arsenic is mainly metabolised by methylation in the liver[5]; however, the methylation of arsenic is not exactly a detoxification process. Arsenic methylation is regulated by glutathione (GSH). The toxicity of monomethylarsonic acid $(MMA)^{3+}$ produced by arsenic metabolism is much higher than that of inorganic arsenic (iAs). Different metabolism levels of arsenic methylation may be an important reason for arsenic-induced liver damage. Lipid peroxidation caused by oxidative stress is considered one of the important mechanisms of arsenic poisoning[6]. Therefore, antagonising the toxicity of arsenic through antioxidation has become an important breakthrough in the prevention and control of arsenic poisoning. Nuclear factor E2-related factor (Nrf2), which is regulated by kelch-like ECH-associated protein-1 (Keap1), is an important regulatory factor of cell resistance to oxidative stress[7]. Nrf2 can regulate various antioxidant enzymes, such as glutathione-S-transferase (GST), heme oxygenase 1 (HO-1), NADPH: quinone oxido-reductase-1 (NQO1), and γ-glutamate-cysteine ligase (γ-GCL)[8], to improve the antioxidant capacity of the body.

Proanthocyanidins can effectively remove various reactive oxygen species (ROS) and have many biological activities, such as antioxidant, free radical scavenging[9], anti-tumour, cardiovascular protective, and cell proliferative effects[10]. Up till now, there are many health products containing proanthocyanidins in the domestic and foreign markets, such as proanthocyanidins capsule and grape seed antioxidant[11]. Proanthocyanidins can be added to yogurt, cakes and other foods as a food supplement. In addition, proanthocyanidins are widely used in cosmetics, such as hand cream and sunscreen cream[12].

Many animal experiments data[13-15] have shown that proanthocyanidins could decrease the content of malondialdehyde (MDA), increase the levels of antioxidant enzymes, such as

glutathione peroxidase (GSH-PX), catalase (CAT) and superoxide dismutase (SOD), and alleviate the oxidative damage induced by zearalenone, aflatoxin B1 and lead in mice. Cell experiments[16, 17] and clinical trials[18, 19] have also shown that proanthocyanidins can effectively reduce oxidative product levels and increase the total antioxidant capacity (T-AOC). Proanthocyanidins, as a natural and efficient free radical scavenger and antioxidant, can help the body to resist lipid peroxidation induced by environmental factors. On one hand, oxidative stress is an important reason for arsenic-induced liver injury. On the other hand, it can regulate cytotoxicity caused by arsenic methylation metabolism.

Grape seed proanthocyanidins extract (GSPE) has high antioxidant activity. However, it is not clear whether proanthocyanidins antagonise arsenic toxicity through Nrf2 pathway in L-02 cells. In addition, the effects of proanthocyanidins on arsenic methylation metabolism have not yet been reported. In our study, L-02 cells were treated with arsenic and/or GSPE for 24 h. We evaluated the antagonistic effect of proanthocyanidins on liver cell toxicity induced by arsenic and explored the possible mechanism by which proanthocyanidins antagonised arsenic-induced oxidative damage of hepatocytes. The findings of this study provide a basis for the development and utilisation of grape seed procyanidin resources and a reference for the prevention and control of endemic arsenic poisoning in Xinjiang.

2. Materials and Methods

2.1. Materials and reagents

Standards of iAs^{3+} ($NaAsO_2$), iAs^{5+}, sodium methyl arsenate, and sodium dimethylarsinate were purchased from Sigma-Aldrich Co. (San Francisco, USA). Commercially available GSPE powder was obtained from Solarbio Science & Technology Co., Ltd. (Beijing, China; purity $\geq$ 95%). Human hepatocytes (L-02 cells) were purchased from Obio Technology Co. Ltd. (Shanghai, China).

SOD, GSH, MDA, sulfhydryl (-SH), alanine aminotransferase (ALT), aspartate aminotransferase (AST), and ROS kits were purchased from Nanjing Jiancheng Bioengineering Institute (Nanjing, China). The kits for MRP1, SAM, As3MT, and AQP3 were purchased from Elabscience Biotechnology Co., Ltd. (Wuhan, China). Anti-Nrf2, anti-HO-1, anti-NQO1, and anti-GST antibodies were obtained from Abcam Ltd. (Abcam, Cambridge, UK). ML385 (Lot No.:

846577-71-9), an Nrf2 inhibitor, was purchased from MedChemExpress (New Jersey, USA). Dulbecco's modified Eagle's medium (DMEM), hyperglycaemic medium, foetal bovine serum (FBS), and trypsin were all purchased from Gibco (California, USA).

2.2. Cell culture

L-02 cells were cultured in DMEM containing 100 U/mL penicillin, 100 mg/mL streptomycin, and 10% (v/v) FBS. The cells were cultured in 25-cm^2 flasks and seeded onto 6-well tissue-culture plates at 37℃ with 5% CO_2. The medium was replaced every 2 days until the cells reached 80–90% confluence (3–5 days). After reaching 80–90% confluence, arsenic (final concentration 0 and 25 μmol/L) and/or GSPE (final concentration 0, 10, 25, and 50 mg/L) were added into the medium at the same time for 24 h. Each experiment was repeated three times.

2.3. Inhibition of Nrf2 expression

ML385[20] was used to inhibit the expression of Nrf2. The concentration of ML385 standard was 10 mmol/L. The medium without penicillin-streptomycin was used to dilute the standard to 2, 4, 6, 8, and 10 μmol/L. L-02 cells were treated with the diluted standard for 48 and 72 h. The optimal intervention concentration and intervention time were screened according to Nrf2 expression, see Figure S1.

2.4. Detection of indicators

2.4.1 MTT viability assay

MTT (3-(4,5-dimethylthiazol-2-yl)-2,5-diphenyltetrazolium bromide) is a tetrazolium dye that is reduced by NAD(P)H-dependent cellular oxidoreductase enzymes, primarily within the mitochondria of viable cells, to yield an insoluble formazan derivative, which can be solubilised and assayed colorimetrically as an indicator of cell viability. L-02 cell line were seeded (about 5×10^3 cells were added at 100μL per well) in 96-well plate. Cells were treated with As or GSPE after adherence at 37ºC and 5% CO_2 atmosphere for 12, 24 and 48 hours. Results were expressed as survival rate, with 100% representing control cells. MTT data were obtained from duplicate wells per treatment shown for three independent experiments.

2.4.2 Annexin V-FITC/propidium iodide apoptosis assay

The percentage of early apoptosis and necrosis was measured using an Annexin V-FITC/propidium iodide (PI) apoptosis kit for flow cytometry, according to manufacturer’s

instructions (Invitrogen, Grand Island, NY, USA). After treatment, the cells were harvested, washed twice with phosphate-buffered saline (PBS), and then incubated with 5 μL FITC-Annexin V and 1 μL PI working solution (100 μg/mL) for 15 min in the dark at 37°C. Cellular fluorescence was measured by flow cytometry analysis using a flow cytometer (FACS Calibur, BD Biosciences, CA, USA).

2.4.3 Measurement of ROS

2',7'-Dichlorodihydrofluorescein diacetate (DCFH-DA) fluorescent labelling was used to measure intracellular ROS production in L-02 cells. For the procedure, L-02 cells were exposed to GSPE (10, 25, and 50 mg/L), arsenic (25 mM), or arsenic + GSPE for 24 h. Then, the cell supernatants were removed and DCFH-DA was added to each group. After incubation with DCFH-DA for 30 min at 37°C, the cells were washed twice with PBS and maintained in 1 mL serum-free medium. The fluorescence images were captured by a fluorescence microscope (OLYMPUS U-RFLT50, Japan) under ×100 magnification, with a filter at excitation and emission wavelengths of 500 and 525 nm, respectively.

2.4.4 Detection of oxidative stress and liver function

GSH specifically deoxidises dithiobisnitrobenzoic acid (DTNB) to form a yellow product 2-nitro-5-SH-benzoic acid, which can be measured by colorimetry at 532 nm. SOD activity was measured using a tetrazolium salt for detection of superoxide radicals generated by xanthine oxidase and hypoxanthine. One unit of SOD is defined as the amount of enzyme needed to exhibit 50% dismutation of the superoxide radical at 37°C. The reaction product was measured at 450 nm. MDA, a marker of lipid peroxidation, was measured with a commercial kit according to the manufacturer's instructions. Briefly, the samples were treated with thiobarbituric acid, which produces a red compound with an absorption maximum at 532 nm in the presence of MDA. The concentration of MDA was calculated by comparing its absorbance to that produced by the standard, 1, 1, 3, 3-tetraethoxypropane. DTNB can react with compounds containing -SH to form a yellow compound.

At 37°C and pH 7.4, ALT and AST can react with alanine and α-ketoglutarate, respectively, to form pyruvic acid and glutamic acid. However, DNPH can terminate the reaction and react with pyruvic acid to form phenylpyruvate, which is rufous under alkaline conditions.

2.4.5 Determination of arsenic methylation

High performance liquid chromatography-hydride generation atomic fluorescence spectra (HPLC-HGAFS) method was used to determine the methylation products of arsenic inside and outside cells, including iAs^{3+}, iAs^{5+}, MMA, and dimethylarsinic acid (DMA). Cells and culture media were collected after completion of cell intervention. After cell disruption, the cells (200μL) were filtered with 0.2μm filtration membrane. Chromatographic eluent was 15mmol/L $(NH_4)_2HPO_4$ solution (pH = 6.0, filtered with 0.45μm fiber membrane before use), and the flow rate was 1.0ml /min. The negative pressure of the photomultiplier tube was 285V. The total current of the hollow cathode lamp was 80mA, the auxiliary current was 36mA. The flow rate of carrier gas was 400ml/min. The current-carrying was 7% hydrochloric acid. The reducing agent included 1.5% KBH_4 solution and 0.35% KOH mixture. The chromatographic column needs to be calibrated for 30 minutes before use. The percentage of iAs^{3+}, iAs^{5+}, MMA, and DMA, as well as primary methylation index (PMI) and secondary methylation index (SMI) were calculated.

PMI=DMA/total arsenic (TAs)

SMI=DMA/(MMA+DMA)

In addition, arsenic methylation results were detected at 12, 24, and 48 h. To evaluate the arsenic methylation capacity of cells at each period, we introduced two new concepts: average growth rate of methylation and methylation average speed in the corresponding period. For example, 12 h–24 h average growth rates of monomethylation = (MMA 24 h+DMA 24 h−MMA 12 h−DMA 12 h)/(TAs 24 h−MMA 12 h−DMA 12 h); 12 h–24 h average growth rates of dimethylation = (DMA 24 h−DMA 12 h)/(MMA 24 h+DMA 24 h−DMA 12 h); 12 h–24 h monomethylation average speed = (MMA 24 h−MMA 12 h+ DMA 24 h−DMA 12 h)/12 h; and 12 h–24 h dimethylation average speed = (DMA 24 h−DMA 12 h)/12 h.

2.4.6 Detection of mRNA of Nrf2-related genes by real-time polymerase chain reaction

Total RNA was extracted by Trizol extraction method (Invitrogen), according to the manufacturer's instructions. Equal amounts of RNA (2 μg) were reverse-transcribed into cDNA using the Transcriptor First-Strand cDNA Synthesis Kit (Roche, Indianapolis, IN, USA). The primers were synthesised by Sigma-Aldrich (St. Louis, MO, USA) for the following genes (Table 1).

Table 1. qRT-PCR primer design

Genes	Size	F	R
Nrf2	155 bp	5′-CCCAGCACATCCAGTCAGAAA-3′	5′-AAACGTAGCCGAAGAAACCTC-3′
HO-1	101 bp	5′-GGCCAGCAACAAAGTGCAAGA-3′	5′-TAAGGACCCATCGGAGAAGCG-3′
NQO1	129 bp	5′-CGCAGACCTTGTGATATTCCAGT-3′	5′-GGTCCTTTGTCATACATGGCAGC-3′
GST	246 bp	5′-AGGACCTTGGATGACTTTCTGA-3′	5′-CACCTTTGGCGTTGCGATCTTTT-3′
β-actin	240 bp	5′-CACGATGGAGGGGCCGGACTCATC-3′	5′-TAAAGACCTCTATGCCAACACAGT-3′

All samples were tested in triplicate[3]. Real-time quantitative polymerase chain reaction (qRT-PCR) was performed on a mixture containing 10 μL PCR Supermix (Bio-Rad Laboratories, Hercules, CA, USA), 1 μL forward and reverse primers (Sangon, Beijing, China), 1 μL template DNA, and 8 μL distilled water. The qPCR conditions were as follows: one cycle of initial denaturation (94°C for 3 min), 30 cycles of amplification (94°C for 30 s, 57°C for 30 s, and 72°C for 25 s), one cycle of melting curve measurement (95°C for 5 s, 65°C for 60 s, and a gradual increase in temperature to 97°C), and a cooling period (40°C for 30 s). The data presented were mRNA levels normalised relative to β-actin.

2.4.7 Detection of protein expression of Nrf2-related proteins by western blotting

L-02 cells were homogenised in one volume of sample buffer [50 mM Tris-Cl, 100 mM DTT, 10% glycerol, and 2% sodium dodecyl sulphate (SDS)] and centrifuged at 14800 ×*g* at 4°C for 15 min to remove debris. The samples were subjected to SDS-polyacrylamide gel electrophoresis and transferred to polyvinylidene difluoride membranes. After blocking with skim milk (5%), the blots were probed with primary antibodies (Abcam, Cambridge, UK) for Nrf2 (1:1000), HO-1 (1:1000), NQO1 (1:1000), GST (1:1000), and β-actin (1:1000) at 4°C for 8 h. Incubation with primary antibodies was followed by incubation with secondary antibodies (conjugated to horseradish peroxidase) after washing in Tris-buffered saline and Tween 20. The blots were processed using an enhanced chemiluminescence (ECL) kit (Santa Cruz Biotechnology, Inc.) and exposed to film. All experiments were repeated three times.

2.5. Statistical analysis

The results were expressed as the mean ± standard deviation. Analysis of variance (ANOVA) was used to detect differences among experimental groups (control, arsenic, GSPE, and arsenic +

GSPE). ANOVA was followed by pairwise comparisons with Bonferroni's multiple comparison tests. The data were analysed using SPSS software for Windows version 17.0 (SPSS Inc., Chicago, IL, USA). A P value < 0.05 was considered statistically significant.

3. Results

3.1. Effects of GSPE and arsenic on cell viability

We first examined the effects of GSPE and arsenic on cell viability by MTT assay. Cell viability decreased significantly with increase in concentration and intervention time of arsenic ($P < 0.05$) when compared with the control group. However, after 12 and 24 h of GSPE intervention, the cell activity did not change significantly ($P > 0.05$) (Figure 1).

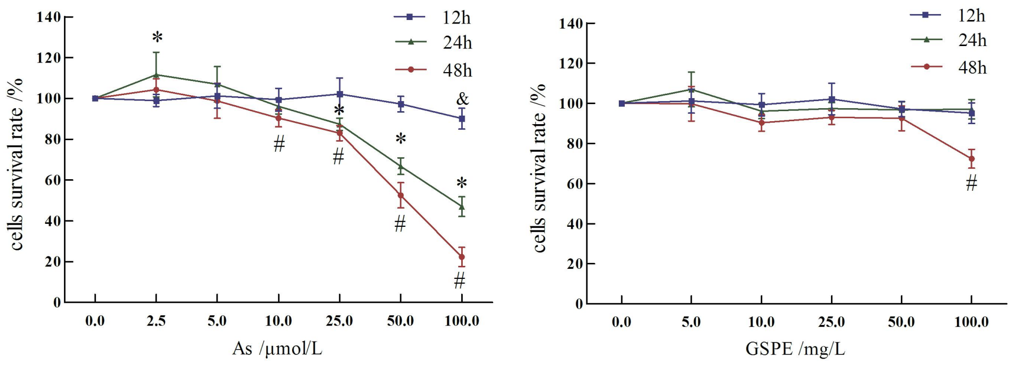

Fig. 1 Viability changes of L-02 cells after arsenic or grape seed procyanidin extract treatment. (Mean & 95% CI). Cell viability was detected by MTT assay. &: 12 h versus control group, $P < 0.05$; *: 24 h, versus control group, $P < 0.05$; #: 48 h, versus control group, $P < 0.05$. CI: Confidence Interval. Cell viability decreased significantly with increase in concentration and intervention time of arsenic. GSPE had no significant effect on cell viability.

To confirm the protective effects of GSPE on arsenic-induced cytotoxicity, cell apoptosis was examined by flow cytometry. GSPE did not induce noticeable apoptosis of L-02 cells [Figure 2 (A2, A3, A4)], while arsenic exposure resulted in significant apoptosis [Figure 2(B1)]. All these changes were mitigated by GSPE [Figure 2(B2, B3, B4)].

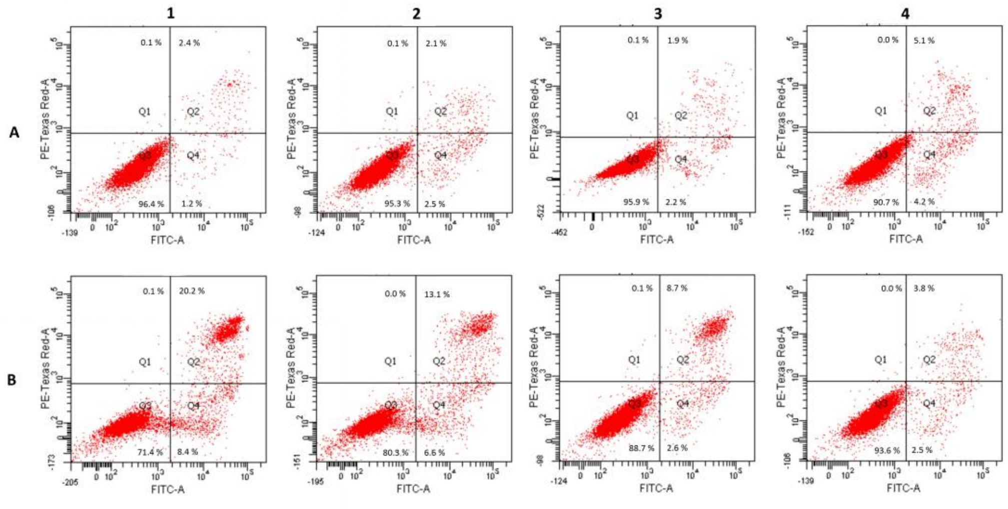

Fig. 2 Effects of arsenic and/or grape seed procyanidin extract on apoptosis. A1: Control group showed normal levels of L-02 cell apoptosis. The percentage of apoptotic cells was less than 5%. A2-A4: GSPE (10, 25, and 50 mg/L for 24 h) treatment groups showed normal L-02 cell apoptosis compared with the control group. In arsenic (25 μM for 24 h) treatment group, apoptosis increased significantly and was mainly advanced apoptosis (20.2%). B2-B4: arsenic (25 μM) + GSPE (10, 25, and 50 mg/L) group (for 24 h) showed gradual decrease in apoptosis with increase in GSPE concentrations.

3.2. GSPE alleviated oxidative stress and liver damage induced by arsenic

In our study, we found that ROS fluorescence intensity was significantly higher in arsenic group (Figure 3E) than in the control group (Figure 3A). However, GSPE effectively removed ROS induced by arsenic (Figure 3F, 3G, 3H). Further quantitative detection of ROS in cells was performed by flow cytometry and similar results were obtained (Figure 4).

We tested the activities of ALT and AST in culture medium and found that ALT and AST activities in arsenic group were markedly higher than those in the control group ($P < 0.05$). Arsenic + GSPE treatment decreased the activities of ALT and AST compared with the arsenic group (Figure 5).

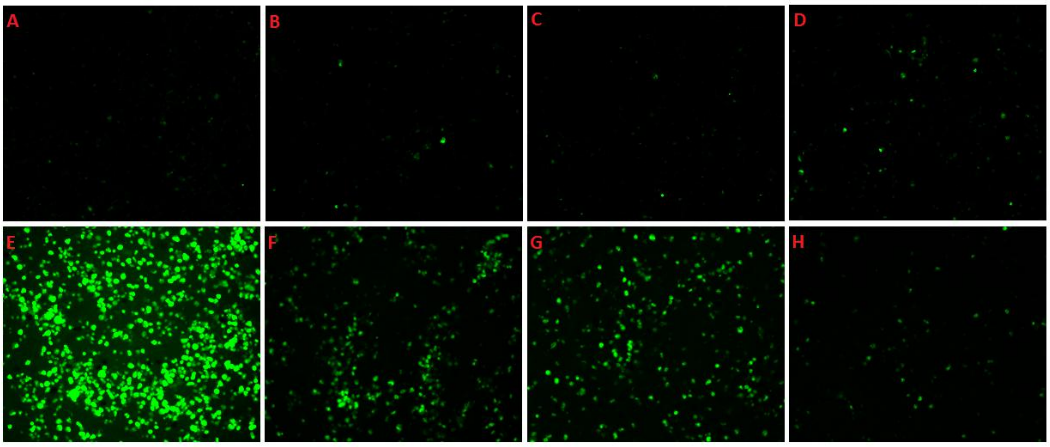

Fig. 3 ROS fluorescence intensity in L-02 cells. A: Control group showed weak ROS fluorescence intensity ; B-D: GSPE (10, 25, and 50 mg/L for 24 h) groups showed weaker fluorescence intensity than the control group; E: Intense green fluorescence of ROS was found in arsenic (25 μM) treatment group. F-H: arsenic (25 μM) + GSPE (10, 25, and 50 mg/L) group (for 24 h) displayed gradual decrease in fluorescence intensity with increase in GSPE concentrations. ROS fluorescence intensity was significantly higher in arsenic group (Figure 3E) than in the control group (Figure 3A). However, GSPE effectively removed ROS induced by arsenic (Figure 3F, 3G, 3H).

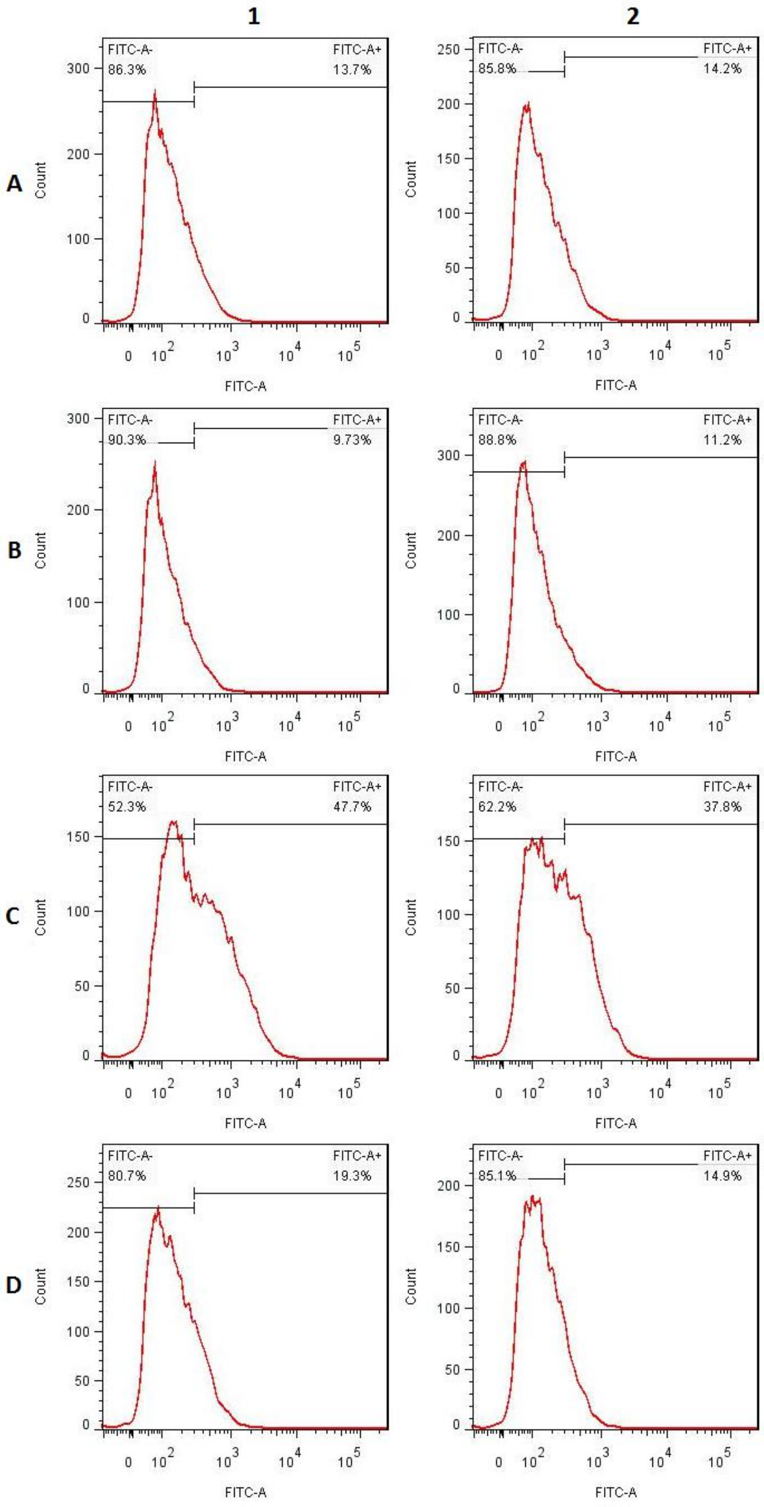

Fig. 4 ROS changes in L-02 cells after arsenic and/or grape seed procyanidin extract treatment for 24 h. The more the number of FITC-A$^+$ cells, the more the ROS in the cells. A1: control group; A2: 10 mg/L GSPE group; B1: 25 mg/L GSPE group; B2: 50 mg/L GSPE group; C1: arsenic (25 μM) group; C2: arsenic (25 μM) + 10 mg/L GSPE group; D1: arsenic (25 μM) + 25 mg/L GSPE group; D2: arsenic (25 μM) + 50 mg/L GSPE group.

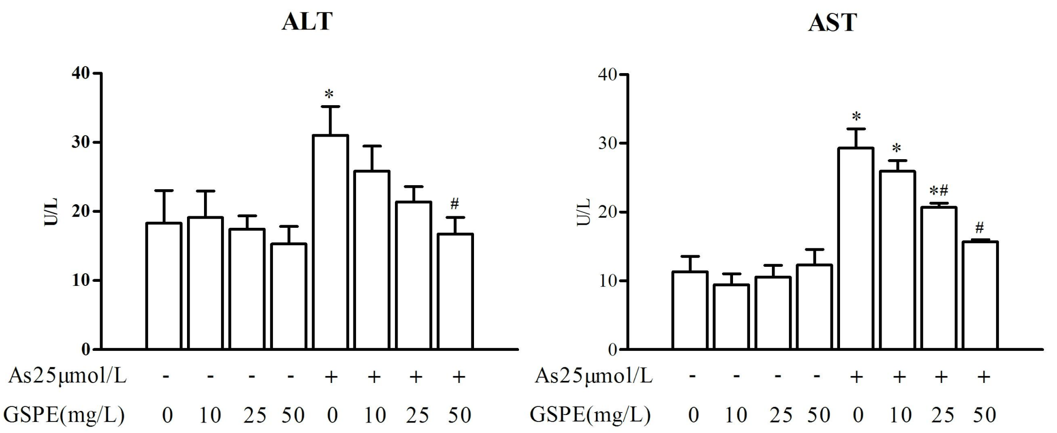

Fig. 5 Effects of arsenic and/or grape seed procyanidin extract on liver function after 24 h (mean ± SD, n = 3). *: versus control group, P < 0.05; #: versus arsenic group, P < 0.05. ALT and AST activities in arsenic group were markedly higher than those in the control group. Arsenic + GSPE treatment decreased the activities of ALT and AST compared with the arsenic group.

We used arsenic as an exogenous oxidative stressor in cells. See Figure 6. We tested GSH, SOD, and -SH levels in cells to assess the effects of arsenic on the antioxidant system. Treatment with arsenic caused a prominent decrease in GSH, SOD, and -SH levels compared with the control group ($P < 0.05$). MDA content was significantly higher in arsenic group than in control group ($P < 0.05$). As shown in Figure 6, treatment with arsenic + GSPE elevated GSH, -SH, and SOD levels in L-02 cells compared with the arsenic group. In addition, arsenic + GSPE group had a lower MDA content than the arsenic group. Hence, GSPE reduced oxidative stress induced by arsenic.

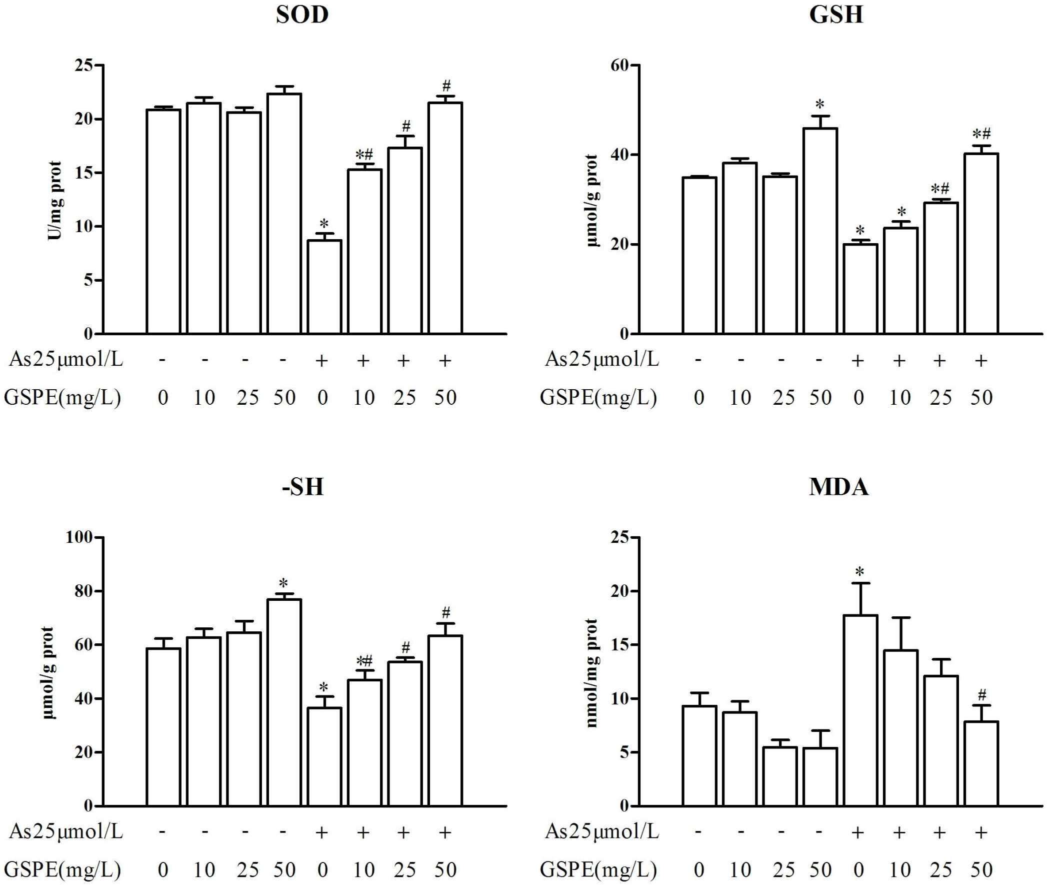

Fig. 6 Oxidative stress changes in L-02 cells after arsenic and/or grape seed procyanidin extract treatment for 24 h (mean ± SD, n = 3). *: versus control group, P < 0.05; #: versus arsenic group, P < 0.05. Treatment with arsenic caused a prominent decrease in GSH, SOD, and -SH levels compared with the control group. MDA content was significantly higher in arsenic group than in control group. Treatment with arsenic + GSPE elevated GSH, -SH, and SOD levels compared with the arsenic group.

3.3. Effects of GSPE on arsenic methylation

To investigate the effects of GSPE on arsenic methylation, we examined the contents of different valence states of arsenic in L-02 cells, including iAs^{3+}, iAs^{5+}, MMA, and DMA. Treatment with 50 mg/L GSPE caused a marked decrease in iAs^{3+} levels compared with arsenic group (P < 0.05). However, MMA content was significantly higher in 25 and 50 mg/L GSPE groups than in arsenic group ($P < 0.05$). DMA content in each GSPE group was higher than that in arsenic group ($P < 0.05$; Figure 7).

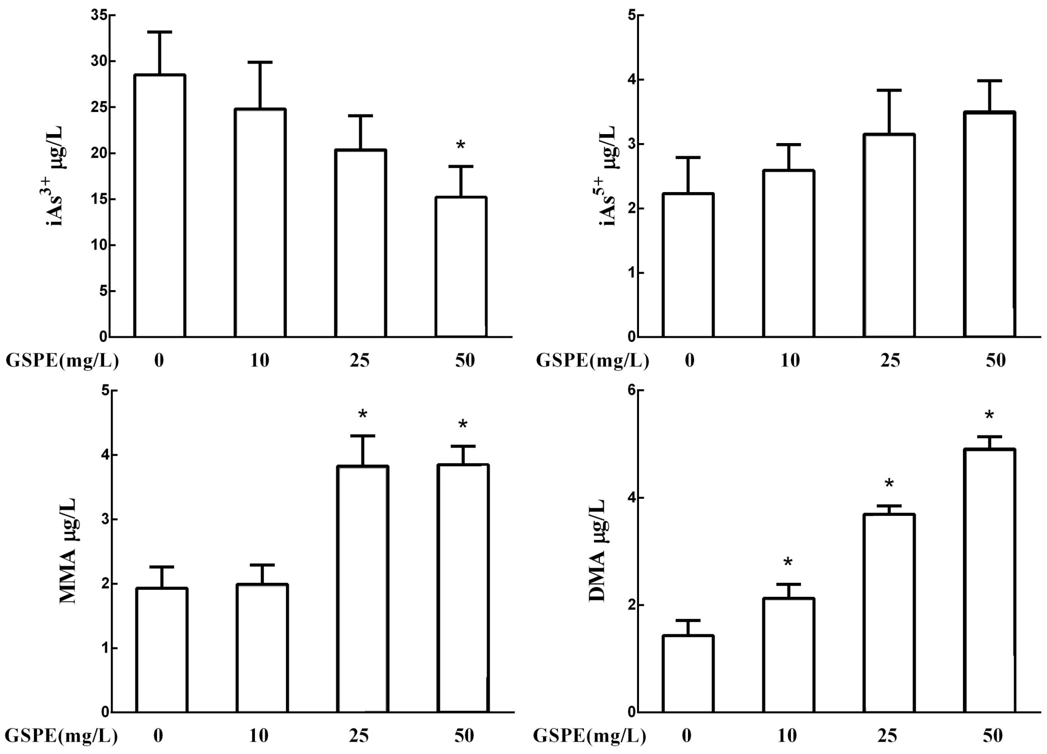

Fig. 7 Effect of grape seed procyanidin extract on arsenic methylation (mean ± SD, n = 3). *: versus arsenic only group, $P < 0.05$. Treatment with 50 mg/L GSPE caused a marked decrease in iAs^{3+} levels compared with arsenic group. However, MMA content was significantly higher in 25 and 50 mg/L GSPE groups than in arsenic group. DMA content in each GSPE group was higher than that in arsenic group.

Furthermore, the proportions of various arsenic compounds in cells and medium were calculated. Compared with the GSPE untreated group, 25 and 50 mg/L GSPE reduced the proportion of iAs3+ ($P < 0.05$) and increased the proportion of iAs5+, MMA, and DMA ($P < 0.05$) in cells. Similarly, treatment with GSPE for 24 h increased the proportion of DMA in medium compared with the untreated group (Figure 8). PMI and SMI were calculated, and the results showed that GSPE significantly increased PMI and SMI in a dose-dependent manner ($P < 0.05$; Table 2).

Table 2. Effect of grape seed proanthocyanidins extract on primary methylation index and secondary methylation index

Indices	GSPE（mg/L）			
	0	10	25	50
PMI	2.12±0.25	3.25±0.27*	3.89±0.23*	5.06±0.40*
SMI	32.22±1.10	48.30±0.98*	52.70±0.75*	55.28±0.42*

*: *P* < 0.05 versus GSPE untreated group.

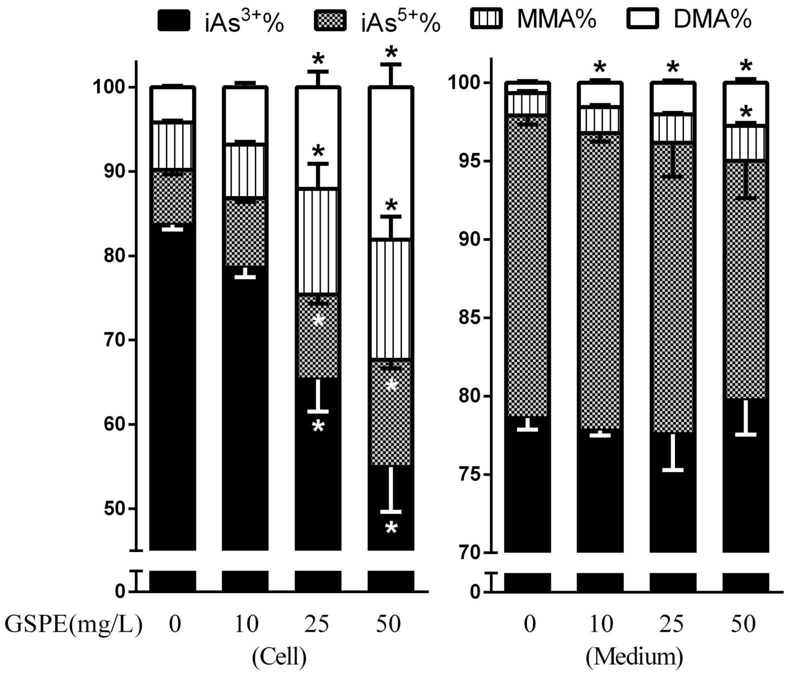

Fig. 8 Proportion of arsenic methylated metabolites in cells and medium (mean ± SD, n = 3). The cells were treated with arsenic (25 μM) and grape seed procyanidin extract (GSPE; 10, 25, and 50 mg/L) for 24 h. *: $P < 0.05$ compared with GSPE untreated group. Compared with the GSPE untreated group, 25 and 50 mg/L GSPE reduced the proportion of iAs^{3+} and increased the proportion of iAs^{5+}, MMA, and DMA in cells. Similarly, treatment with GSPE for 24 h increased the proportion of DMA in medium compared with the untreated group.

To explore the effect of GSPE on arsenic methylation further, the average growth rate of methylation and average methylation speed were calculated. We found that the average growth rates of monomethylation and dimethylation in GSPE treatment groups at 12, 24, and 48 h were higher than those in GSPE untreated group ($P < 0.05$; Figure 9A, 9B). Furthermore, GSPE promoted the average methylation speed of monomethylation and dimethylation at 12, 24, and 48 h compared with GSPE untreated group (Figure 9C, 9D).

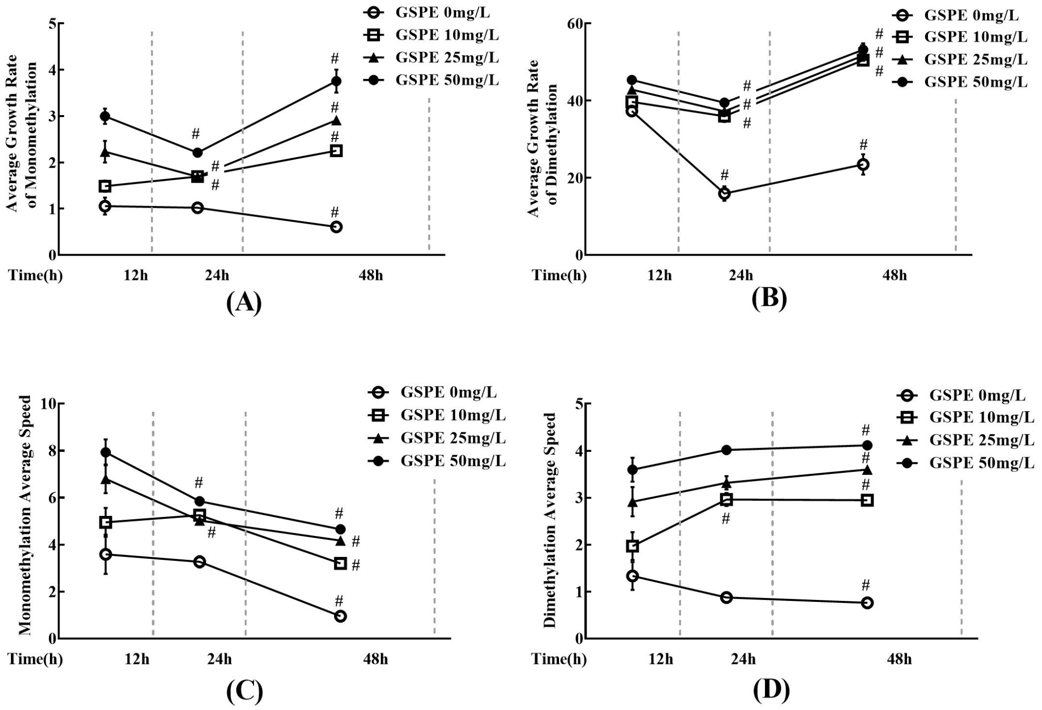

Fig. 9 Effects of grape seed procyanidin extract on average growth rate of methylation and average methylation speed (mean ± SD, n = 3). #: versus 0 ‑ 12 h groups at the same dose of GSPE, $P < 0.05$. The average growth rates of monomethylation and dimethylation in GSPE treatment groups at 12, 24, and 48 h were higher than those in GSPE untreated group (Figure 9A, B). Furthermore, GSPE promoted the average methylation speed of monomethylation and dimethylation at 12, 24, and 48 h compared with GSPE untreated group (Figure 9C, D).

3.4. Changes in Nrf2 signal pathway

L-02 cells were treated with arsenic (25 μM) and GSPE (10, 25, and 50 mg/L) for 24 h, and the mRNA and protein expression of Nrf2 and its target genes were detected by qRT-PCR and western blotting. We found that treatment with GSPE (25 and 50 mg/L) significantly elevated the protein contents of Nrf2, NQO1, HO-1, and GST in a dose-dependent manner compared with the control group ($P < 0.05$). Compared with the control group, arsenic (25 μM) markedly decreased the protein expression of Nrf2 and its downstream genes after 24 h ($P < 0.05$). However, the protein contents of Nrf2, NQO1, HO-1, and GST in arsenic + GSPE group increased gradually compared with the contents in arsenic group (Figures 10 and 11). Similar trends were observed for mRNA expression of Nrf2 and its downstream genes (Table 3).

Table 3. mRNA expression of Nrf2 and its downstream genes

Indices	Control	GSPE (mg/L)			As (μmol/L)	As+GSPE		
		10	25	50	25	10	25	50
Nrf2	1.00±0.00	1.19±0.20	1.34±0.27	1.69±0.35*	0.41±0.02*	0.77±0.04	0.99±0.12#	1.04±0.11#
HO-1	1.00±0.00	1.36±0.20	2.37±0.36*	2.64±0.28*	0.44±0.10*	0.66±0.27	1.03±0.34#	2.17±0.12*#
NQO1	1.00±0.00	1.57±0.02*	2.21±0.24*	2.67±0.22*	0.50±0.16*	0.62±0.11	1.00±0.18#	1.35±0.07#
GST	1.00±0.00	1.24±0.05	1.57±0.10*	1.64±0.12*	0.40±0.16*	0.55±0.08*	1.12±0.07#	1.45±0.10*#

Note: the results were described as mean ± SD (n = 3). * indicates significant difference from control group at $P < 0.05$; # indicates significant difference from arsenic group at $P < 0.05$.

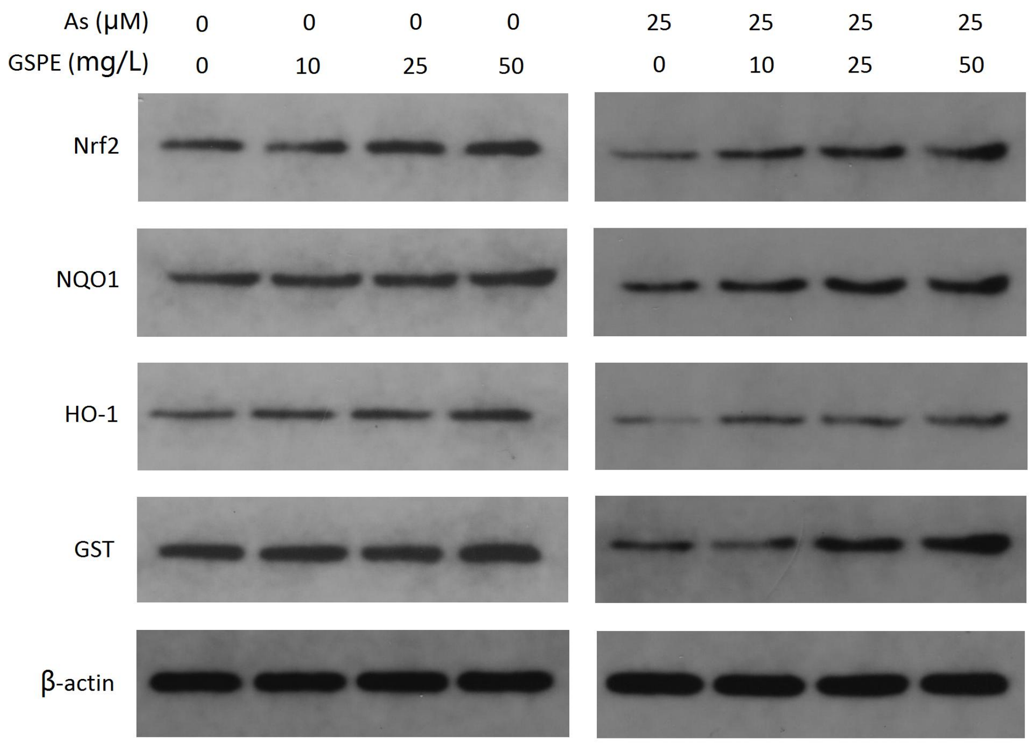

Fig. 10 Western blot analysis of proteins after treatment with arsenic and/or grape seed procyanidin extract. Nrf2, NQO1, HO-1, and GST protein expression was measured in L-02 cells treated with arsenic and/or grape seed procyanidin extract by western blotting. Treatment with GSPE (25 and 50 mg/L) significantly elevated the protein contents of Nrf2, NQO1, HO-1, and GST in a dose-dependent manner compared with the control group. Compared with the control group, arsenic (25 μM) markedly decreased the protein expression of Nrf2 and its downstream genes after 24 h. However, the protein contents of Nrf2, NQO1, HO-1, and GST in arsenic + GSPE group increased gradually compared with the contents in arsenic group.

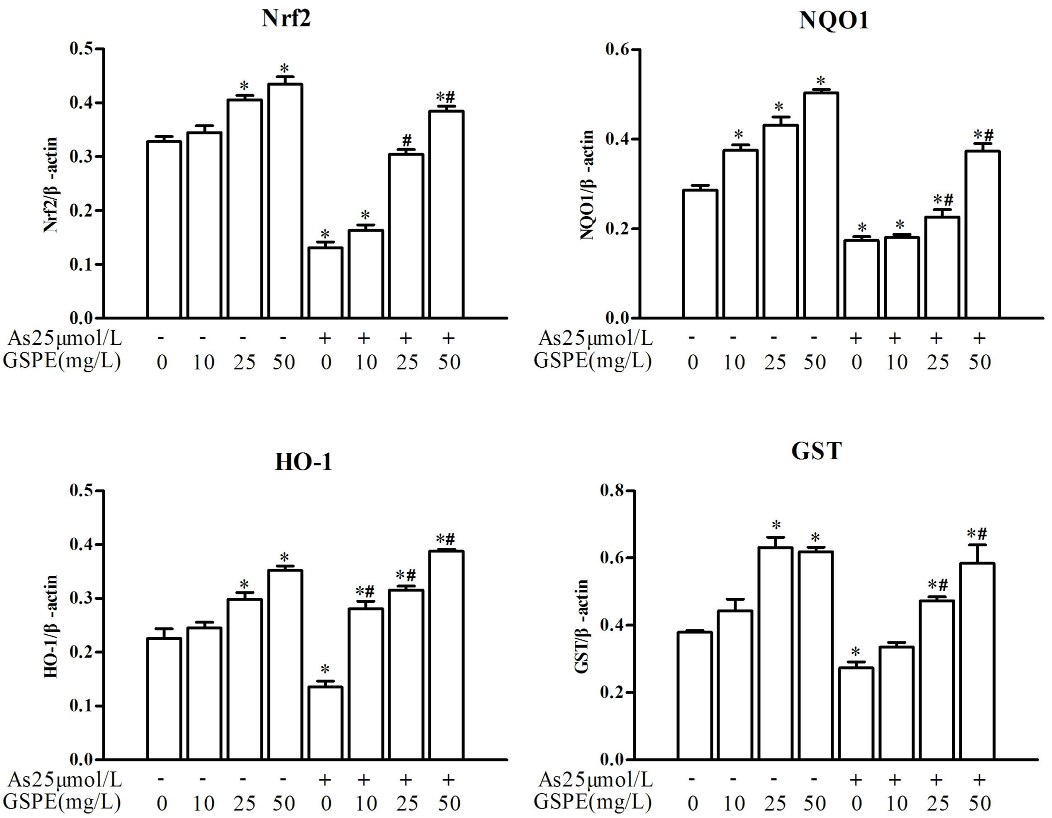

Fig. 11 Quantitative protein analysis after treatment with arsenic and/or grape seed procyanidin extract. Nrf2, NQO1, HO-1, and GST protein expression levels were measured. Y-axis represents protein expression of test protein relative to β-actin. Each bar represents the mean ± SD. Significant differences relative to the control and arsenic groups are indicated as follows: *: versus control group, $P < 0.05$; #: versus arsenic group, $P < 0.05$.

3.5. Changes in liver function and oxidative stress after Nrf2 inhibition

ML385 (5 μM) was used to inhibit the expression of Nrf2. ALT and AST levels in the arsenic group after Nrf2 inhibition were significantly higher than those in the control group ($P < 0.05$) and higher than those in the arsenic group before Nrf2 inhibition ($P < 0.05$). After Nrf2 inhibition, high-dose GSPE could still reduce ALT and AST levels compared with arsenic group, but the reducing effect of GSPE on arsenic-induced ALT and AST levels was weak (Figure 12).

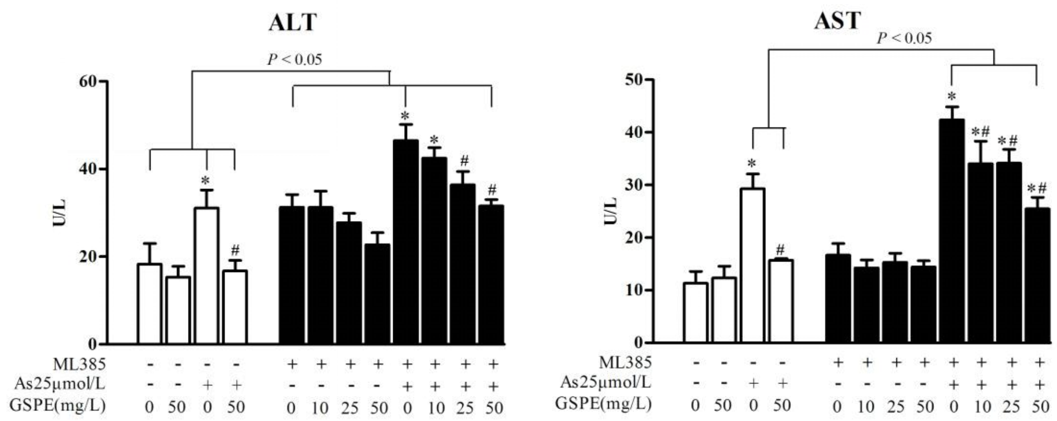

Fig. 12 Effects of arsenic and/or grape seed procyanidin extract on liver function after Nrf2 inhibition. After Nrf2 inhibition, L-02 cells were treated with arsenic and/or grape seed procyanidin extract for 24 h. *: compared with control group, $P < 0.05$; #: compared with arsenic group, $P < 0.05$. After Nrf2 inhibition, high-dose GSPE could still reduce ALT and AST levels compared with arsenic group, but the reducing effect of GSPE on arsenic-induced ALT and AST levels was weak.

Oxidative stress indicators were detected after Nrf2 inhibition. We found that 50 mg/L GSPE could increase SOD activity and reduce MDA level compared with the control group ($P < 0.05$). SOD, GSH, and -SH levels in the arsenic group decreased further after Nrf2 inhibition ($P < 0.05$) when compared with the control group and were lower than those in the arsenic group before Nrf2 inhibition ($P < 0.05$). MDA content increased further after Nrf2 inhibition ($P < 0.05$) and was higher than that in the arsenic group before Nrf2 inhibition ($P < 0.05$). SOD and -SH levels in arsenic + GSPE group increased after Nrf2 inhibition ($P < 0.05$) compared with the levels in the arsenic group, but were still lower than the levels in the arsenic + GSPE group before Nrf2 inhibition ($P < 0.05$; Figure 13).

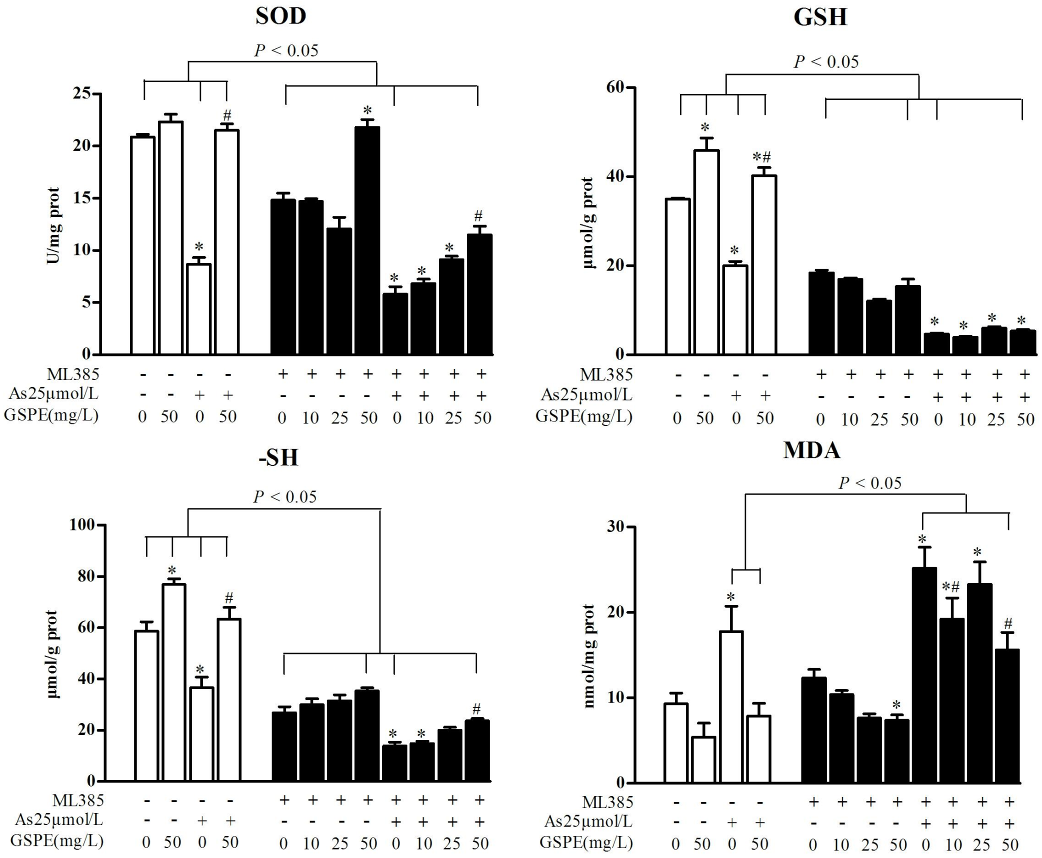

Fig. 13 Effects of arsenic and/or grape seed procyanidin extract on oxidative stress after Nrf2 inhibition. After Nrf2 inhibition, L-02 cells were treated with arsenic and/or grape seed procyanidin extract for 24 h. *: compared with control group, $P < 0.05$; #: compared with arsenic group, $P < 0.05$. 50 mg/L GSPE could increase SOD activity and reduce MDA level compared with the control group. SOD, GSH, and -SH levels in the arsenic group decreased further after Nrf2 inhibition when compared with the control group and were lower than those in the arsenic group before Nrf2 inhibition. MDA content increased further after Nrf2 inhibition and was higher than that in the arsenic group before Nrf2 inhibition. SOD and -SH levels in arsenic + GSPE group increased after Nrf2 inhibition compared with the levels in the arsenic group, but were still lower than the levels in the arsenic + GSPE group before Nrf2 inhibition.

3.6. Role of Nrf2 in arsenic methylation

After Nrf2 inhibition, treatment with 50 mg/L GSPE caused a prominent increase in iAs^{3+} levels compared with the same intervention in Nrf2 normal cells (P < 0.05). However, DMA content in 50 mg/L GSPE group after Nrf2 inhibition was lower than that in Nrf2 normal cells treated with the same dose ($P < 0.05$). In Nrf2-suppressed cells, GSPE had no significant effect on MMA compared with Nrf2 normal cells ($P > 0.05$). In addition, MMA content in Nrf2-suppressed cells

was higher than that in Nrf2 normal cells ($P < 0.05$) (Figure 14).

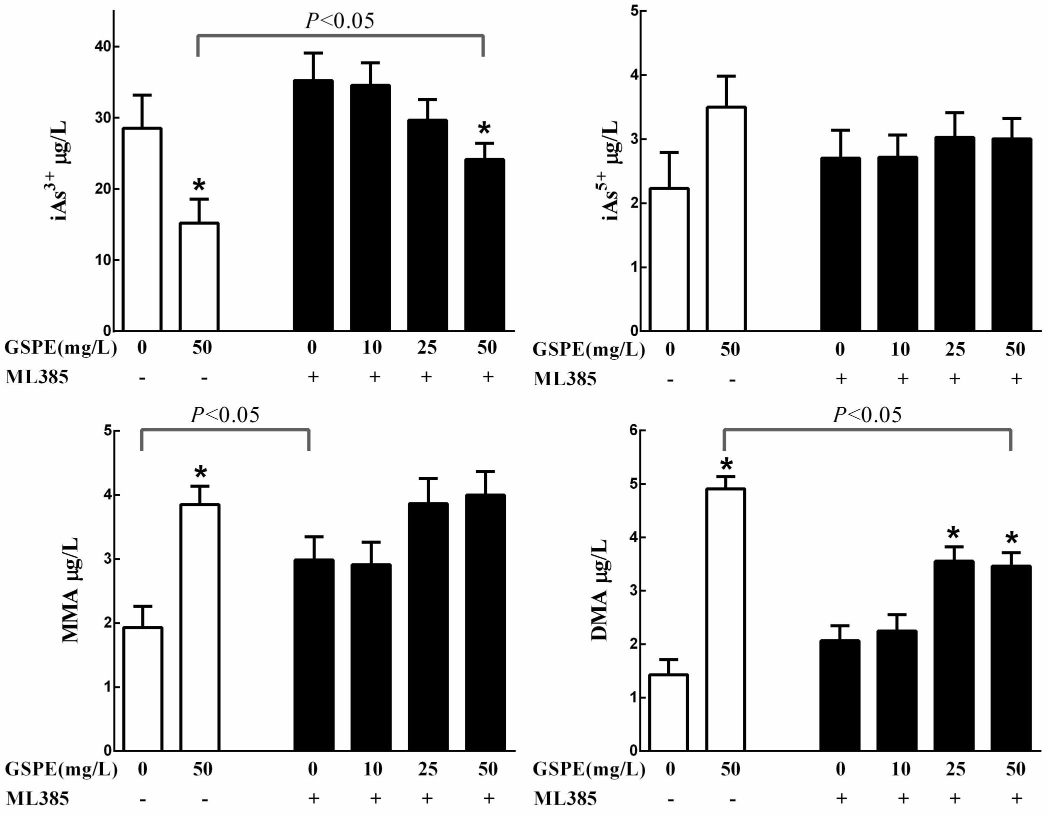

Fig. 14 Effects of grape seed procyanidin extract on arsenic methylation in Nrf2-suppressed cells (mean ± SD, n = 3). Nrf2-suppressed cells were treated with arsenic (25 μM) and grape seed procyanidin extract (GSPE; 0, 10, 25, and 50 mg/L) for 24 h. *: versus GSPE (0 mg/L) group, $P < 0.05$. Treatment with 50 mg/L GSPE caused a prominent increase in iAs^{3+} levels compared with the same intervention in Nrf2 normal cells. However, DMA content in 50 mg/L GSPE group after Nrf2 inhibition was lower than that in Nrf2 normal cells treated with the same dose. In Nrf2-suppressed cells, GSPE had no significant effect on MMA compared with Nrf2 normal cells. In addition, MMA content in Nrf2-suppressed cells was higher than that in Nrf2 normal cells.

Furthermore, the proportions of various arsenic compounds in Nrf2-suppressed cells and its medium were calculated. The iAs^{3+} proportion was higher in Nrf2-suppressed cells treated with GSPE (50 mg/L) than in Nrf2 normal cells treated with the same dose ($P < 0.05$). However, the DMA proportions in Nrf2-suppressed cells treated with GSPE (50 mg/L) and in medium were lower than that in Nrf2 normal cells and medium treated with the same dose ($P < 0.05$) (Figure 15).

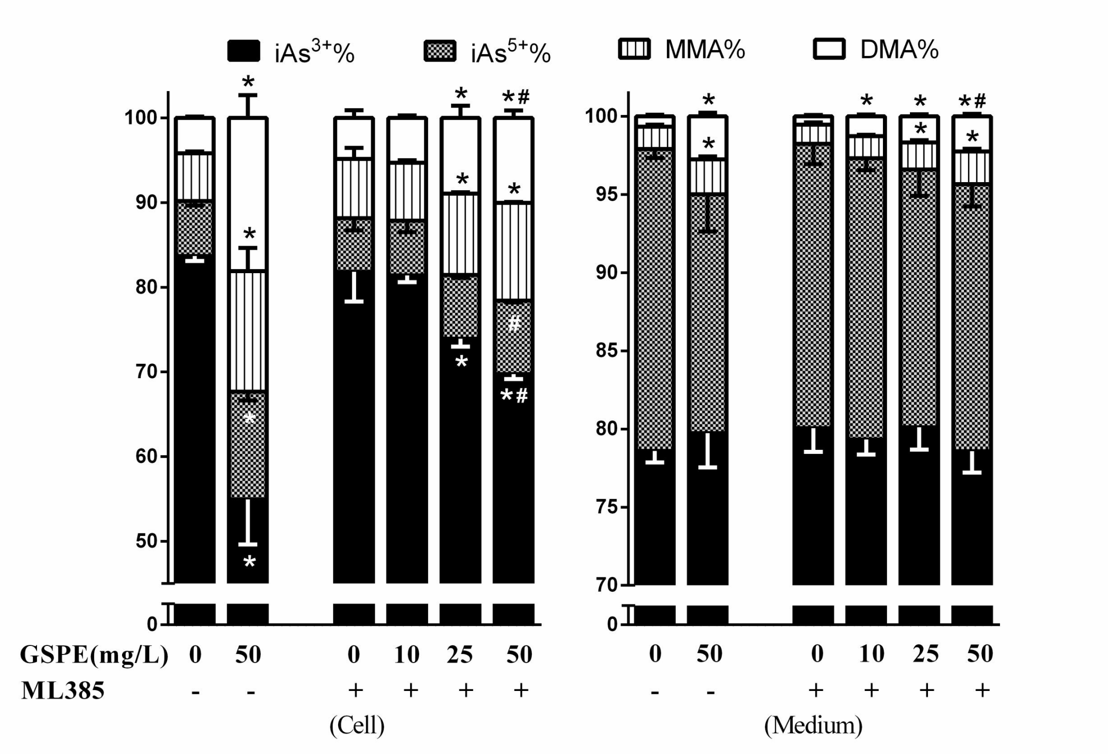

Fig. 15 Proportion of arsenic compounds in Nrf2-suppressed cells (mean ± SD, n = 3). Nrf2-suppressed cells were treated with arsenic (25 μM) and grape seed procyanidin extract (GSPE; 0, 10, 25, and 50 mg/L) for 24 h. *: versus GSPE (0 mg/L) group, $P < 0.05$; #: versus the same intervention group in Nrf2 normal cells, $P < 0.05$. The proportions of various arsenic compounds in Nrf2-suppressed cells and its medium were calculated. The iAs^{3+} proportion was higher in Nrf2-suppressed cells treated with GSPE (50 mg/L) than in Nrf2 normal cells treated with the same dose. However, the DMA proportions in Nrf2-suppressed cells treated with GSPE (50 mg/L) and in medium were lower than that in Nrf2 normal cells and medium treated with the same dose.

Table 4 shows that the SMI was lower in Nrf2-suppressed cells treated with GSPE (50 mg/L) than in Nrf2 normal cells treated with the same dose ($P < 0.05$). The average growth rate of methylation and average methylation speed in Nrf2-suppressed cells were calculated. We found that between 0–12 h, the average growth rate of dimethylation in Nrf2-suppressed cells of each group cells was lower than that in Nrf2 normal cells treated with the same doses ($P < 0.05$). Between 12–24 h, the average growth rate of monomethylation in each GSPE group and the average growth rate of dimethylation of GSPE (25 and 50 mg/L) group were lower ($P < 0.05$) in Nrf2-suppressed cells than in Nrf2 normal cells treated with the same doses. Between 24–48 h, the average growth rate of monomethylation in each GSPE group and the average growth rate of dimethylation of GSPE (50 mg/L) group were lower ($P < 0.05$) in Nrf2-suppressed cells than in

Nrf2 normal cells treated with the same doses (Figure 16A, 16B).

Table 4. Effects of grape seed proanthocyanidins extract on primary methylation index and secondary methylation index in Nrf2-suppressed cells

GSPE (mg/L)		Nrf2 normal cells		Nrf2-suppressed cells			
		0	50	0	10	25	50
PMI	Mean	2.12	5.06*	1.80	2.71*	3.45*	4.40*
	SD	0.25	0.40	0.22	0.20	0.28	0.32
SMI	Mean	32.22	55.28*	31.02	47.63*	49.43*	51.63*#
	SD	1.10	0.42	0.44	0.88	0.40	0.15

*: versus GSPE (0 mg/L) group, $P < 0.05$; #: versus the same intervention group in Nrf2 normal cells, $P < 0.05$.

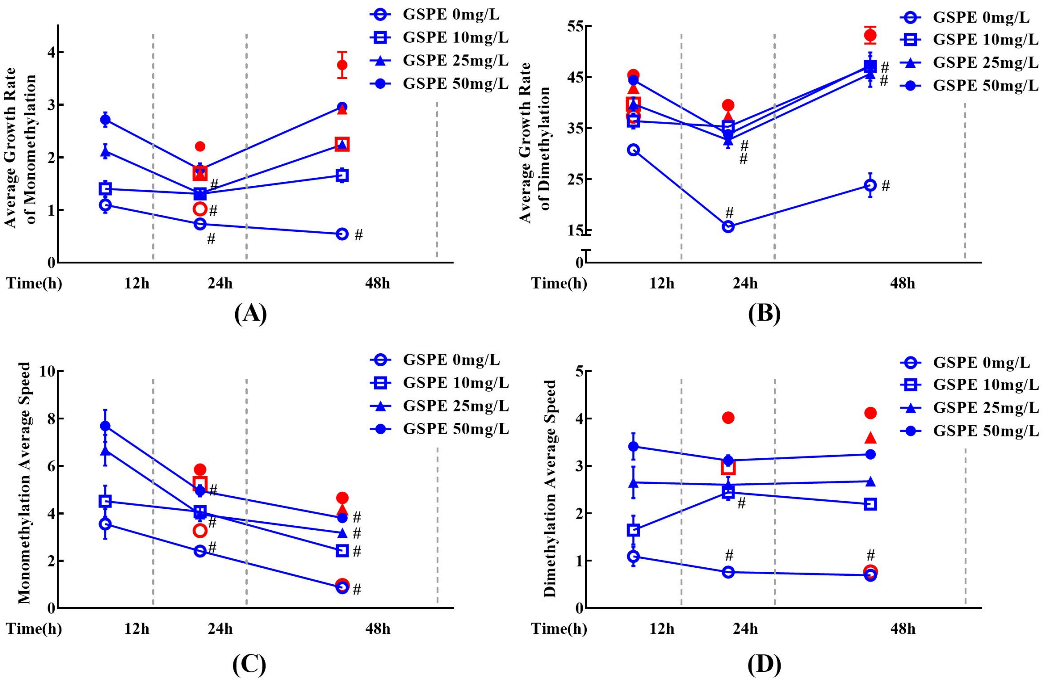

Fig. 16 Effects of grape seed procyanidin extract on average growth rate of methylation and average methylation speed in Nrf2-suppressed cells (mean ± SD, n = 3). Nrf2-suppressed cells were treated with arsenic (25 μM) and grape seed procyanidin extract (GSPE; 0, 10, 25, and 50 mg/L) for 24 h. #: versus 0–12 h group, $P < 0.05$; red shapes indicate the level in Nrf2 normal cells versus the same intervention group in Nrf2-suppressed cells, $P < 0.05$.

We calculated the average methylation speed of monomethylation and dimethylation as well. Between 12–24 h, the average methylation speed of monomethylation and dimethylation in Nrf2-suppressed cells treated with GSPE (10 and 50 mg/L) was lower than that in Nrf2 normal

cells treated with the same doses ($P < 0.05$). Between 24–48 h, the average methylation speed of monomethylation and dimethylation in Nrf2-suppressive cells treated with GSPE (25 and 50 mg/L) was lower than that in Nrf2 normal cells treated with the same doses ($P < 0.05$; Figure 16C, 16D).

3.7 Effects of GSPE on Nrf2 pathway after Nrf2 inhibition

The mRNA expression of Nrf2 and its downstream genes significantly decreased after Nrf2 inhibition. In Nrf2-suppressed cells, low-dose GSPE had no significant effect on the mRNA expression of Nrf2 and its downstream genes ($P > 0.05$) compared with the control group. In Nrf2-suppressed cells, GSPE could still mitigate arsenic-induced decrease in mRNA expression of Nrf2 and downstream genes; however, its effect was weaker than that in Nrf2 normal cells (Figure 17). Similary effect was found in the exoression of Nrf2 and its downstream proteins (Figure S2).

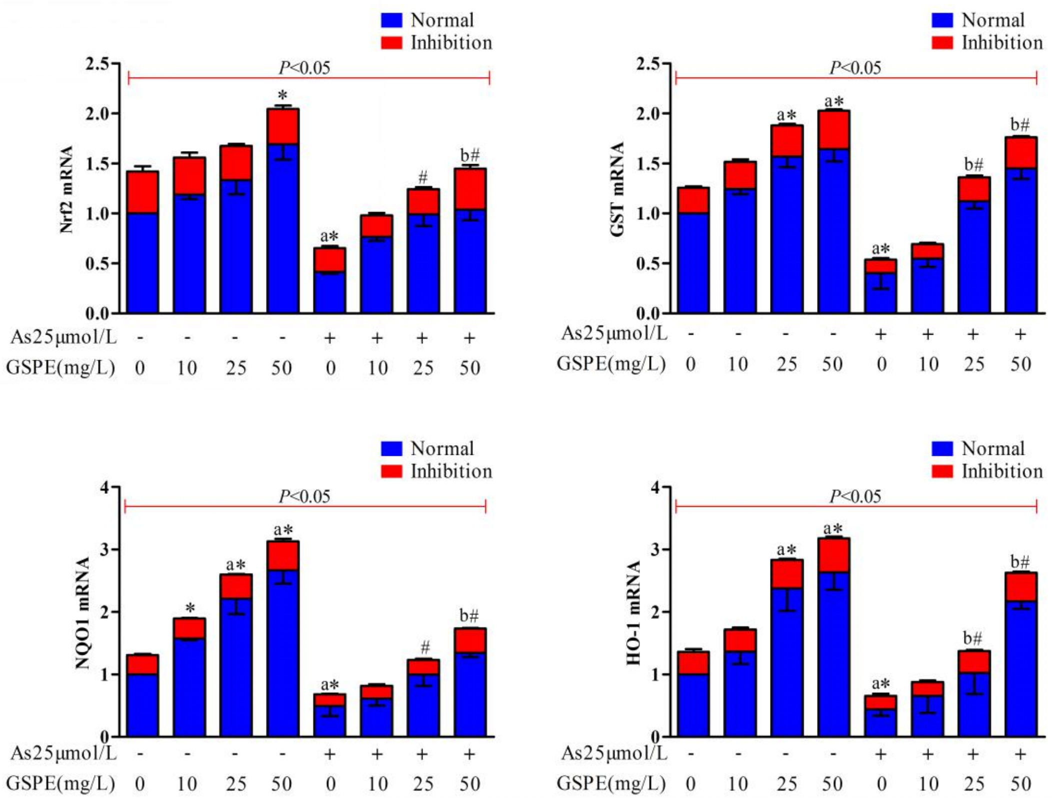

Fig. 17 mRNA expression of Nrf2 pathway in Nrf2-suppressed cells (mean ± SD, n = 3). L-02 cells were treated with arsenic and/or grape seed procyanidin extract (GSPE) for 24 h. *: in Nrf2 normal cells versus control group, $P < 0.05$; #: in Nrf2 normal cells versus arsenic group, $P < 0.05$; a: in Nrf2 inhibition cells versus control group, $P < 0.05$; b: in Nrf2 inhibition cells versus arsenic group, $P < 0.05$. In Nrf2-suppressed cells, GSPE could still mitigate arsenic-induced decrease in mRNA expression of Nrf2 and downstream genes; however, its effect was weaker than that in Nrf2 normal cells.

Discussion

In this study, we aimed to determine the effects of GSPE on oxidative damage and arsenic methylation, and to clarify the role of Nrf2 in the process. We found that GSPE activates Nrf2 signalling pathway to antagonise arsenic-induced oxidative damage and to promote arsenic methylation metabolism.

Arsenic exposure can cause liver fibrosis, cirrhosis, and even liver cancer in severe cases[21]. Therefore, analysing the hepatotoxicity of arsenic and its toxic action mechanism is of great importance for preventing and treating arsenic poisoning. Proanthocyanidins are a type of powerful free radical scavenger and antioxidant found in nature. In this study, arsenic was found to inhibit Nrf2 signalling pathway, which led to oxidative damage of cells and cell apoptosis. Moreover, the cytotoxic effect of arsenic is closely related to its metabolism in cells. GSPE can activate Nrf2 signalling pathway and antagonise arsenic-induced oxidative damage, promote arsenic methylation metabolism, and accelerate the metabolism and excretion of arsenic to reduce cell damage. The results of this experiment also provide the basis and evidences for future studies on the action mechanism of arsenic and proanthocyanidins.

Our study showed that arsenic changed the morphology and structure of cells. Arsenic also decreased cell activity and increased apoptosis. Arsenic is cytotoxic and can cause liver damage, and this aspect is speculated to be related to the fact that arsenic can cause oxidative stress in cells[22, 23]. On the other hand, arsenic may inhibit the expression of Bcl2[24] leading to apoptosis. When proanthocyanidins were treated alone, no significant change in cell activity, apoptosis, and liver function were observed. Therefore, proanthocyanidins have been identified as a safe antioxidant[25]. Moreover, proanthocyanidins could significantly improve cell activity reduction and apoptosis induced by arsenic, indicating that they could antagonise the cytotoxicity of arsenic. These activities are speculated to be related to the antioxidation of proanthocyanidins. Another possibility is that proanthocyanidins promoted the expression of Bcl2 and inhibited apoptosis.

Oxidative stress is one of the action mechanisms of arsenic. Our study proved that arsenic can cause oxidative damage to cells probably via ROS generation[26], antioxidant enzyme reduction[27], and MDA production. In addition, GSH could easily combine with iAs^{3+} and other

trivalent arsenides[28], resulting in the reduction of GSH. Moreover, GSH is used during the methylation process of iAs^{3+}[29]. Arsenic can reduce the expression of Nrf2 and its downstream genes. The reduction of antioxidant enzymes induced by arsenic was more obvious in Nrf2-suppressed cells, indicating that arsenic may inhibit Nrf2 pathway[30, 31] and reduce antioxidant capacity. The mechanism by which arsenic inhibits Nrf2 expression may involve acceleration of Nrf2 degradation by promoting Keap1 protein expression[32]. Proanthocyanidins can improve oxidative damage induced by arsenic[33, 34]. However, the effect of proanthocyanidins on antioxidant enzymes was significantly decreased in Nrf2-suppressed cells. Furthermore, proanthocyanidins could promote the expression of Nrf2 and its downstream genes. It indicated that Nrf2 plays an important role in the action of proanthocyanidins. The underlying mechanism may be through promotion of Nrf2 dissociation from Keap1[35], after which Nrf2 enters the nucleus to combine with antioxidant responsive element (ARE) on DNA[36], which induces the expression of its downstream antioxidant enzymes. Proanthocyanidins contain a large amount of H^+, which can block free radical chain reaction, thus improving the activity of various antioxidant enzymes and antioxidant substances in cells. See Figure 18.

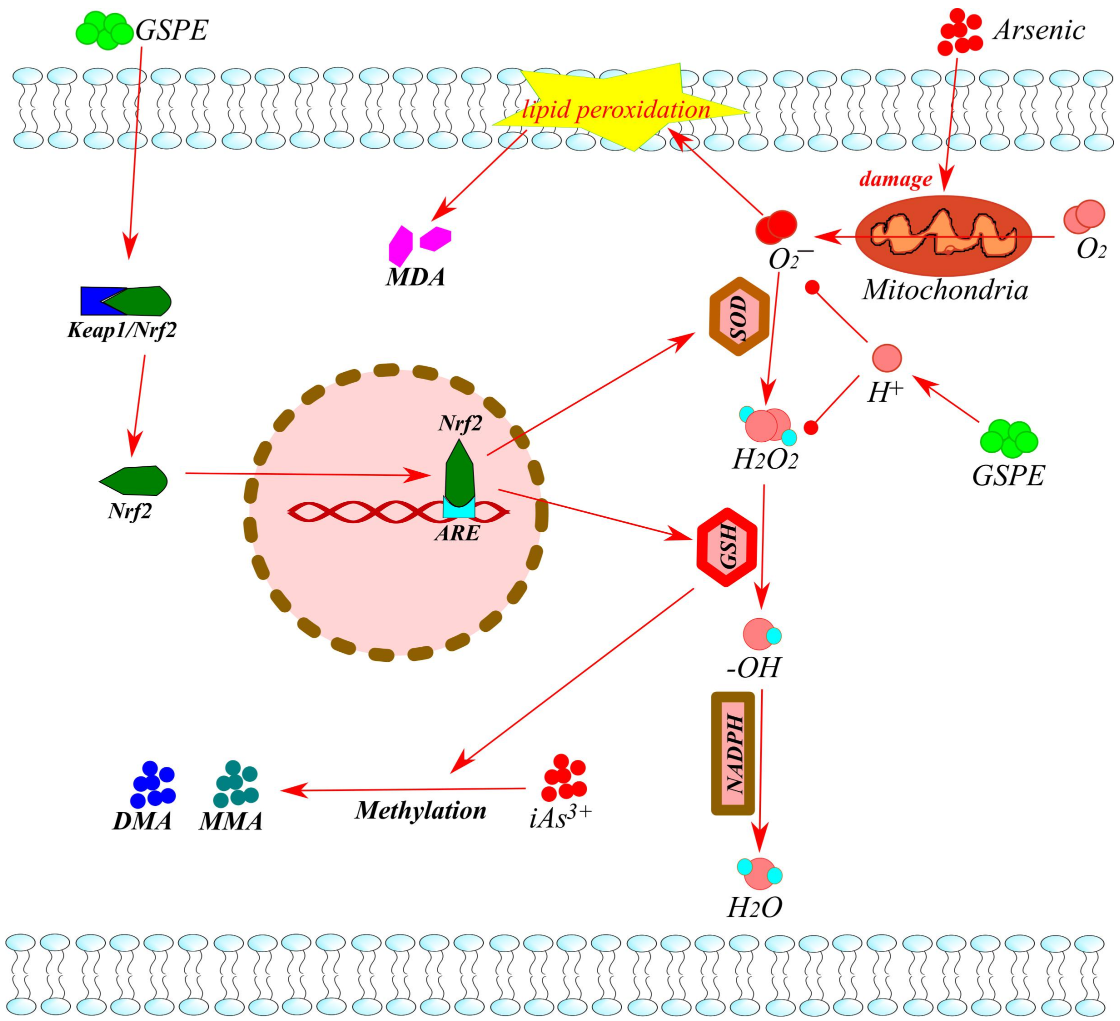

Fig 18. The action mechanism of GSPE on oxidative stress induced by arsenic and methylation of arsenic. Arsenic can cause oxidative injuries by reducing the activity of anti-oxidative enzymes and increasing the amount of peroxide products. GSPE contains many phenolic hydroxyl groups and release H^+, which can bind active oxygen radicals and competitively block the reaction chains of free radicals, reducing the consumption of antioxidants and increasing the activity of antioxidant enzymes. In addition, GSPE promotes the dissociation of Nrf2 and Keap1 proteins, allowing Nrf2 to enter the nucleus from the cytoplasm, thus promoting the expression of downstream genes of Nrf2 and increasing the antioxidant capacity. Nrf2 pathway activation increases the level of GSH, which promotes the methylation of arsenic. (Arrows: promotion; blunt arrows: inhibition).

Arsenic toxicity is also closely related to its metabolism in cells. Trivalent arsenic compounds, which have a high affinity to sulfhydryl, are more easily absorbed by the cells than any other valence states of arsenic. Therefore, trivalent arsenic can inhibit the activity of enzymes that contain sulfhydryl; consequently, cell respiration, division, and proliferation are affected and cell metabolism is disturbed[37, 38]. Epidemiological studies have reported individual differences in

arsenic methylation, and people with weak methylation are more sensitive to arsenic-induced health damage[39]. It has been found that curcumin and sodium mercaptosulfonic acid can promote the metabolism and excretion of arsenic in mouse[40] and Chang liver cells[41], and these effects are considered to be related to the activation of Nrf2 signalling pathway. In our study, we found that GSPE can promote arsenic methylation metabolism in cells and improve the arsenic methylation ability. After Nrf2 inhibition, the cells still showed methylation metabolism ability, and GSPE improved the arsenic metabolism ability of the cell. However, arsenic methylation ability in Nrf2-suppressed cells was inferior to that in normal cells, and the effect of GSPE on arsenic metabolism in Nrf2-suppressed cells was weaker than that in normal cells. In addition, GSPE significantly improved the dimethylation capacity of arsenic compared with the monomethylation ability. These findings suggest that GSPE can promote arsenic methylation metabolism in L-02 cells, improve the arsenic methylation ability of cells, especially dimethylation metabolism, reduce the action time of MMA to cells, and decrease toxicity of arsenic to cells.

At present, endemic arsenism is still a worldwide public health problem, and our study found that proanthocyanins has a good effect on the liver damage caused by arsenic. In addition, proanthocyanins have been widely used in many fields such as medicine, foods and cosmetics, and the safety of proanthocyanins has been well verified[42]. Proanthocyanidins have been used in the adjuvant therapy of patients with obstructive sleep apnea hypopnea syndrome (OSAHS)[43]. Therefore, we believe that proanthocyanins can be used as a potential adjuvant therapy for arsenic poisoning. For example, arsenic poisoning people can eat more fruits riched with proanthocyanins and take some proanthocyanins nourishment while receiving treatment. Procyanidins can also be added to people's daily diet in a right amount.

There is a limitation to this study. ML385 was used to inhibit Nrf2 signalling pathway and reduce the expression of Nrf2 and its downstream genes for exploring the relationship between proanthocyanidins and Nrf2 signalling pathway. Since ML385 is an inhibitor of Nrf2 and not a knockout agent, a small number of Nrf2 genes were still expressed.

In conclusion, proanthocyanidins can activate Nrf2 signalling pathway to antagonise arsenic-induced oxidative damage and to promote arsenic methylation metabolism.

Proanthocyanidins can reduce arsenic toxicity and promote arsenic elimination. Nrf2 signalling pathway plays an important role in the antioxidation process of proanthocyanidins and is of great significance for the prevention and treatment of arsenic poisoning.

Data Availability

The research data used to support the findings of this study are included within the article and the supplementary information file.

Conflict of interest

The authors declare no conflict of interest.

Acknowledgments

The authors would like to thank the Department of Public Health, Shihezi University School of Medicine for assistance with this work, as well as funding from the National Natural Science Foundation of China (No. 81760584; No. 81560517), the Key Areas of Science and Technology Research Project of Xinjiang Production and Construction Corps (No. 2014BA039, No. 2015AG014), Research Innovation Project for Postgraduates of Xinjiang Autonomous Region (No. XJGRI2016053), and the International Cooperation Program of Shihezi University (No. GJHZ201602).

References:

1. Li YF, Ye F, Wang AW, Wang D, Yang BY, Zheng QM, Sun GF, Gao XH (2016) Chronic Arsenic Poisoning Probably Caused by Arsenic-Based Pesticides: Findings from an Investigation Study of a Household. International Journal of Environmental Research and Public Health 133: 1-14. doi:10.3390/ijerph13010133.

2. Li SG, Ding YS, Niu Q, Xu SZ, Pang LJ, Ma RL, Jing MX, Feng GL, Liu JM, Guo SX (2015) Grape Seed Proanthocyanidin Extract Alleviates Arsenic-induced Oxidative Reproductive Toxicity in Male Mice. Biomedical and environmental sciences : BES 28:272-280. doi 10.3967/bes2015.038.

3. Li SG, Xu SZ, Niu Q, Ding YS, Pang LJ, Ma RL, Jing MX, Wang K, Ma XM, Feng GL, Liu JM, Zhang XF, Xiang HL, Li F (2016) Lutein alleviates arsenic-induced reproductive toxicity in male mice via Nrf2 signaling. Hum Exp Toxicol 35:491-500. doi 10.1177/0960327115595682.

4. Wang P, Yuan ZZ, Zhang HT, Huang ZQ (2013) Analysis of arsenic content in drinking water of rural residents in qianguo county, jilin province. Chin J Ctrl Endem Dis 28: 215-216. doi 10.11847/zgggws2014-30-03-38

5. Drobna Z, Walton FS, Harmon AW, Thomas DJ, Styblo M (2010) Interspecies differences in metabolism of arsenic by cultured primary hepatocytes. Toxicol Appl Pharmacol 245:47-56. doi 10.1016/j.taap.2010.01.015.

6. Das S, Joardar S, Manna P, Dua TK, Bhattacharjee N, Khanra R, Bhowmick S, Kalita J, Saha A, Ray S, De Feo V, Dewanjee S (2018) Carnosic Acid, a Natural Diterpene, Attenuates Arsenic-Induced Hepatotoxicity via Reducing Oxidative Stress, MAPK Activation, and Apoptotic Cell Death Pathway. Oxid Med Cell Longev 2018:1421438. doi 10.1155/2018/1421438.

7. Hu Y, Yu C, Yao ML, Wang L, Liang B, Zhang BX, Huang XX, Zhang AH (2018) The PKCδ-Nrf2-ARE signalling pathway may be involved in oxidative stress in arsenic-induced liver damage in rats. Environ Toxicol Pharmacol 62:79-87. doi: 10.1016/j.etap.2018.05.012.

8. Yang SH, Yu LH, Li L, Guo Y, Zhang Y, Long M, Li P, He JB (2018) Protective Mechanism of Sulforaphane on Cadmium-Induced Sertoli Cell Injury in Mice Testis via Nrf2/ARE Signaling Pathway. Molecules 23. doi 10.3390/molecules23071774.

9. Melo PS, Arrivetti LOR, Alencar SM, Skibsted LH (2016) Antioxidative and prooxidative effects in food lipids and synergism with alpha-tocopherol of acai seed extracts and grape rachis extracts. Food chemistry 213:440-449. doi 10.1016/j.foodchem.2016.06.101.

10. Li SG, Xu MC, Niu Q, Xu SZ, Ding YS, Yan YZ, Guo SX, Li F (2015) Efficacy of Procyanidins against In Vivo Cellular Oxidative Damage: A Systematic Review and Meta-Analysis. PLoS One 10:e0139455. doi 10.1371/journal.pone.0139455.

11. Zhang H, Zeng Q (2011) Research Progress Functions and Applications of Proanthocyanidin. Journal of Anhui Agri. Sci. 39: 5349-5350.

12. Chen MY, Huang XD, Wang Z, Long DF, Zhang K, Wang Y (2018) Research Progress and Application of Plant Proanthocyanidin. Food and Nutrition in China 24: 54-58.

13. Long M, Liu Y, Cao Y, Wang N, Dang M, He JB (2016) Proanthocyanidins attenuation of chronic lead-induced liver oxidative damage in kunming mice via the Nrf2/ARE pathway. Nutrients 8: 656. doi: 10.3390/nu8100656.

14. Long M, Yang SH, Han JX, Li P, Zhang Y, Dong S, Chen X, Guo J, Wang J, He JB (2016) The Protective Effect of Grape-Seed Proanthocyanidin Extract on Oxidative Damage Induced by Zearalenone in Kunming Mice Liver. Int J Mol Sci, 17: E808. doi: 10.3390/ijms17060808.

15. Long M, Zhang Y, Li P, Yang SH, Zhang WK, Han JX, Wang Y, He JB (2016) Intervention of

Grape Seed Proanthocyanidin Extract on the Subchronic Immune Injury in Mice Induced by Aflatoxin B1. Int J Mol Sci, 17: 516. doi: 10.3390/ijms17040516.
16. Zhang T, Jiang S, He C, Kimura Y, Yamashita Y, Ashida H (2013) Black soybean seed coat polyphenols prevent B(a)P-induced DNA damage through modulating drug-metabolizing enzymes in HepG2 cells and ICR mice. Mutation research 752:34-41. doi 10.1016/j.mrgentox.2013.01.002.
17. Dai N, Zou Y, Zhu L, Wang HF, Dai MG (2014) Antioxidant properties of proanthocyanidins attenuate carbon tetrachloride (CCl4)-induced steatosis and liver injury in rats via CYP2E1 regulation. Journal of medicinal food 17:663-669. doi 10.1089/jmf.2013.2834.
18. Peng L, Zhao P, Li B, Zhang JH, Wang YW, Huang CP (2011) Experimental study on the antioxidative function of grape seed powder in human body. CHINA TROPICAL MEDICINE 11: 874-875. doi 10.13604/j.cnki.46-1064/r.2011.07.015
19. Wan Y, Vinson JA, Etherton TD, Proch J, Lazarus SA, Kris-Etherton PM (2001) Effects of cocoa powder and dark chocolate on LDL oxidative susceptibility and prostaglandin concentrations in humans. The American journal of clinical nutrition 74:596-602. doi 10.1093/ajcn/74.5.596.
20. Singh A, Venkannagari S, Oh KH, Zhang YQ, Rohde JM, Liu L, Nimmagadda S, Sudini K, Brimacombe KR, Gajghate S, Ma J, Wang A, Xu X, Shahane SA, Xia M, Woo J, Mensah GA, Wang Z, Ferrer M, Gabrielson E, Li Z, Rastinejad F, Shen M, Boxer MB, Biswal S (2016) Small Molecule Inhibitor of NRF2 Selectively Intervenes Therapeutic Resistance in KEAP1-Deficient NSCLC Tumors. ACS chemical biology 11:3214-3225. doi 10.1021/acschembio.6b00651.
21. Santra A, Maiti A, Das S, Lahiri S, Charkaborty SK, Mazumder DN (2000) Hepatic damage caused by chronic arsenic toxicity in experimental animals. Journal of toxicology. Clinical toxicology 38:395-405. doi 10.1081/CLT-100100949.
22. Shen H, Niu Q, Xu MC, Rui DS, Xu SZ, Feng GL, Ding YS, Li SG, Jing MX (2016) Factors Affecting Arsenic Methylation in Arsenic-Exposed Humans: A Systematic Review and Meta-Analysis. Int J Environ Res Public Health 13:205. doi 10.3390/ijerph13020205.
23. Xu MC, Rui DS, Yan YZ, Xu SZ, Niu Q, Feng GL, Wang Y, Li SG, Jing MX (2017) Oxidative Damage Induced by Arsenic in Mice or Rats: A Systematic Review and Meta-Analysis. Biol Trace Elem Res 176:154-175. doi 10.1007/s12011-016-0810-4.
24. Zhu WH, Xu N, Xu JJ, Zhang H, Li Y, Lv SJ, Zhang W (2018) Effects of dihydroartemisinin on proliferation and apoptosis of human pancreatic cancer cell line. ACTA ANATOMICA SINICA 49: 70-74. doi 10.16098/j.issn.0529-1356.2018.01.011.
25. Mao X (2016) Study on extraction of proanthocyanidins from grape vine and its antioxidant activity. XinJiang University.
26. Kumar S, Yedjou CG, Tchounwou PB (2014) Arsenic trioxide induces oxidative stress, DNA damage, and mitochondrial pathway of apoptosis in human leukemia (HL-60) cells. J Exp Clin Cancer Res 33:42. doi 10.1186/1756-9966-33-42.
27. Singh MK, Yadav SS, Gupta V, Khattri S (2013) Immunomodulatory role of Emblica officinalis in arsenic induced oxidative damage and apoptosis in thymocytes of mice. BMC Complement Altern Med 13:193. doi 10.1186/1472-6882-13-193.
28. Pawlik-Skowronska B, Pirszel J, Kalinowska R, Skowronski T (2004) Arsenic availability, toxicity and direct role of GSH and phytochelatins in As detoxification in the green alga Stichococcus bacillaris. Aquatic toxicology 70:201-212. doi 10.1016/j.aquatox.2004.09.003.
29. Wang S, Li X, Song X, Geng Z, Hu X, Wang Z (2012) Rapid equilibrium kinetic analysis of arsenite methylation catalyzed by recombinant human arsenic (+3 oxidation state)

methyltransferase (hAS3MT). The Journal of biological chemistry 287:38790-38799. doi 10.1074/jbc.M112.368050.
30. Shafik NM, El Batsh MM (2016) Protective Effects of Combined Selenium and Punica granatum Treatment on Some Inflammatory and Oxidative Stress Markers in Arsenic-Induced Hepatotoxicity in Rats. Biol Trace Elem Res 169:121-128. doi 10.1007/s12011-015-0397-1.
31. Vineetha RC, Archana V, Binu P, Arathi P, Nair RH (2018) L-Ascorbic Acid and alpha-Tocopherol Reduces Hepatotoxicity Associated with Arsenic Trioxide Chemotherapy by Modulating Nrf2 and Bcl2 Transcription Factors in Chang liver Cells. Nutrition and cancer 70:684-696. doi 10.1080/01635581.2018.1460676.
32. Ugun-Klusek A, Tatham MH, Elkharaz J, Constantin-Teodosiu D, Lawler K, Mohamed H, Paine SM, Anderson G, John Mayer R, Lowe J, Ellen Billett E, Bedford L (2017) Continued 26S proteasome dysfunction in mouse brain cortical neurons impairs autophagy and the Keap1-Nrf2 oxidative defence pathway. Cell death & disease 8:e2531. doi 10.1038/cddis.2016.443.
33. Liu QQ, Gan ZL, Xie HP, Wang CM, Hao LY, Yang Y (2017) Effects of Proanthocyanidins Resveratrol Pulp on SOD Activity and MDA Content in Rats with Acute Alcohol-induced Hepatic Injury. Food Research And Development 38: 183-186. doi 10.3969/j.issn.1005-6521.2017.19.040.
34. Liu P, Zhou YS, Qin YL, Li L, Liu Y, Xu B, Huang K, Ji CC, Lin F, Wang YG, Li K, Chen SH, Shao LF, Mu JS (2017) Mechanism of action for oligomeric proanthocyaniclins in pava qnat-induced acute lung injury. Chin J Ind Hyg Occup Dis 35: 818-822. doi 10.3760/cma.j.issn.1001-9391.2017.11.004.
35. O'Connell MA, Hayes JD (2015) The Keap1/Nrf2 pathway in health and disease: from the bench to the clinic. Biochemical Society transactions 43:687-689. doi 10.1042/BST20150069.
36. Liu B, Zhang H, Tan X, Yang D, Lv Z, Jiang H, Lu J, Baiyun R, Zhang Z (2017) GSPE reduces lead-induced oxidative stress by activating the Nrf2 pathway and suppressing miR153 and GSK-3beta in rat kidney. Oncotarget 8:42226-42237. doi 10.18632/oncotarget.15033.
37. Li JX, Shen YQ, Cai BZ, Zhao J, Bai X, Lu YJ, Li XQ (2010) Arsenic trioxide induces the apoptosis in vascular smooth muscle cells via increasing intracellular calcium and ROS formation. Mol Biol Rep 37:1569-1576. doi 10.1007/s11033-009-9561-z.
38. Chen BW, Hua N, Lǜ ML, Le XC (2009) Metabolism, Toxicity , and Biomonitoring of Arsenic Species. PROGRESS IN CHEMISTRY 21: 474-482.
39. Li X, Li B, Xi S, Zheng Q, Wang D, Sun G (2013) Association of urinary monomethylated arsenic concentration and risk of hypertension: a cross-sectional study from arsenic contaminated areas in northwestern China. Environmental health : a global access science source 12:37. doi 10.1186/1476-069X-12-37.
40. Gao S, Duan X, Wang X, Dong D, Liu D, Li X, Sun G, Li B (2013) Curcumin attenuates arsenic-induced hepatic injuries and oxidative stress in experimental mice through activation of Nrf2 pathway, promotion of arsenic methylation and urinary excretion. Food Chem Toxicol 59:739-747. doi 10.1016/j.fct.2013.07.032.
41. Duan X, Liu D, Xing X, Li J, Zhao S, Nie H, Zhang Y, Sun G, Li B (2014) Tert-butylhydroquinone as a phenolic activator of Nrf2 antagonizes arsenic-induced oxidative cytotoxicity but promotes arsenic methylation and detoxication in human hepatocyte cell line. Biol Trace Elem Res 160:294-302. doi 10.1007/s12011-014-0042-4.
42. Ling ZQ, Zhang XH, Xie BJ, Zeng FD (2002) Review on the pharmacological research of procyanidins. Chinese Pharmacological Bulletin, 18: 9-12. doi: 10.3321/j.issn:1001-1978.2002.01.004.

43. Li LQ, Fan YH, Wang HY, Guo X (2016) Effect of grape seed proanthocyanidin adjuvant therapy on sleep quality of OSAHS patients. Shandong Medical Journal, 56: 24-27. doi: 10.3969/j.issn.1002-266X.2016.48.007.
