# Supplementary material for: Retracted: Proanthocyanidins Antagonize Arsenic-Induced Oxidative Damage and Promote Arsenic Methylation through Activation of the Nrf2 Signaling Pathway
Source: Oxid Med Cell Longev. 2021 Jan 22;2021:3547620. doi: 10.1155/2021/3547620 (PMC7846395; doi:10.1155/2021/3547620)
Supplement: Supplementary Materials — Figure duplication in Figure 2 of OMCL/8549035. (Supplementary Materials.docx). Corrected figure files (Supplementary Materials.rar). [file 3547620.f1.zip › 3547620.f1/raw data/Duplicated FACS images in panels in Figure 2 of Xu et al OMCL 8549035 (1).docx]

Duplicated FACS images in panels in Figure 2 of Mengchuan Xu, Qiang Niu, Yunhua Hu, Gangling Feng, Haixia Wang, and Shugang Li, “Proanthocyanidins Antagonize Arsenic-Induced Oxidative Damage and Promote Arsenic Methylation through Activation of the Nrf2 Signaling Pathway,” Oxidative Medicine and Cellular Longevity, vol. 2019, Article ID 8549035, 19 pages, 2019. <https://doi.org/10.1155/2019/8549035>

**Figure 2A(1) Figure 2B(4)**


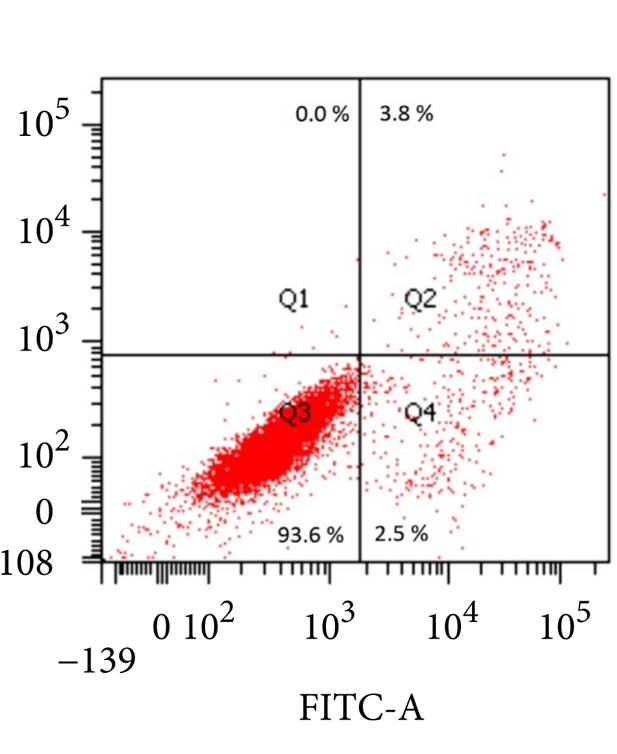

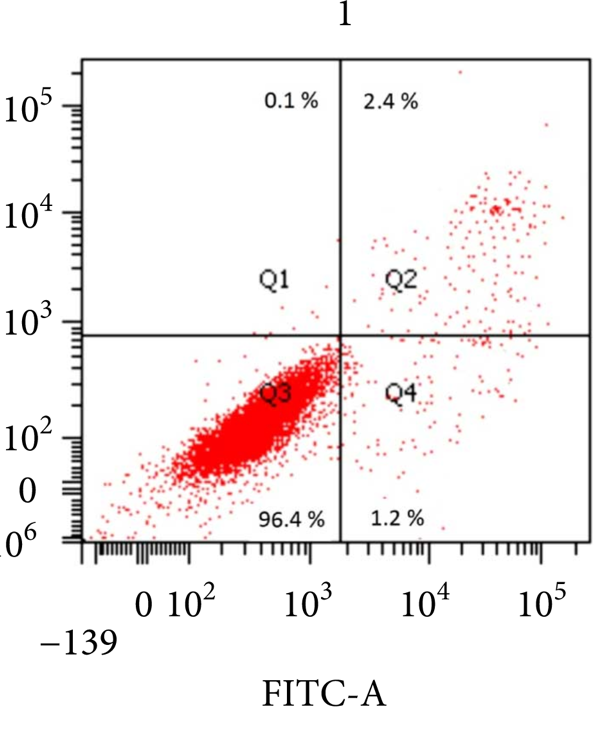


**
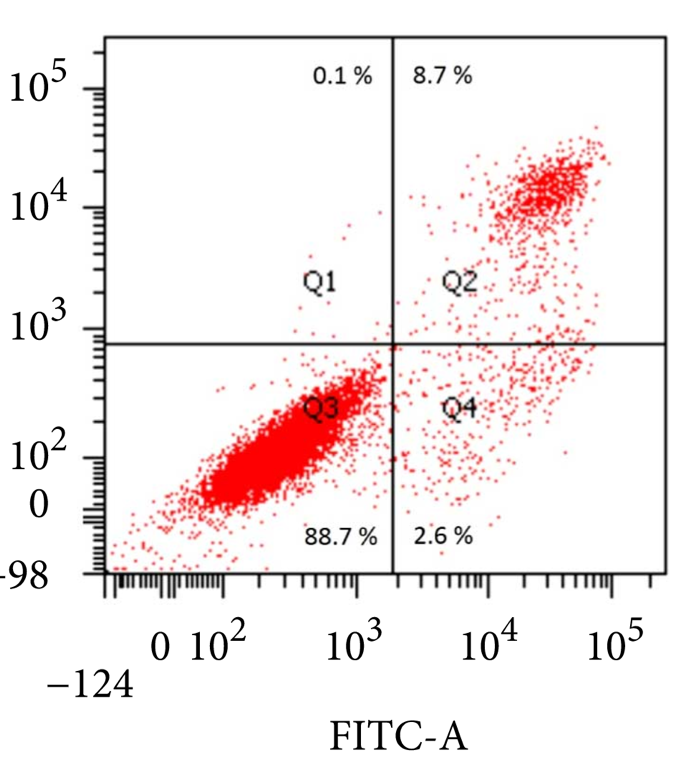

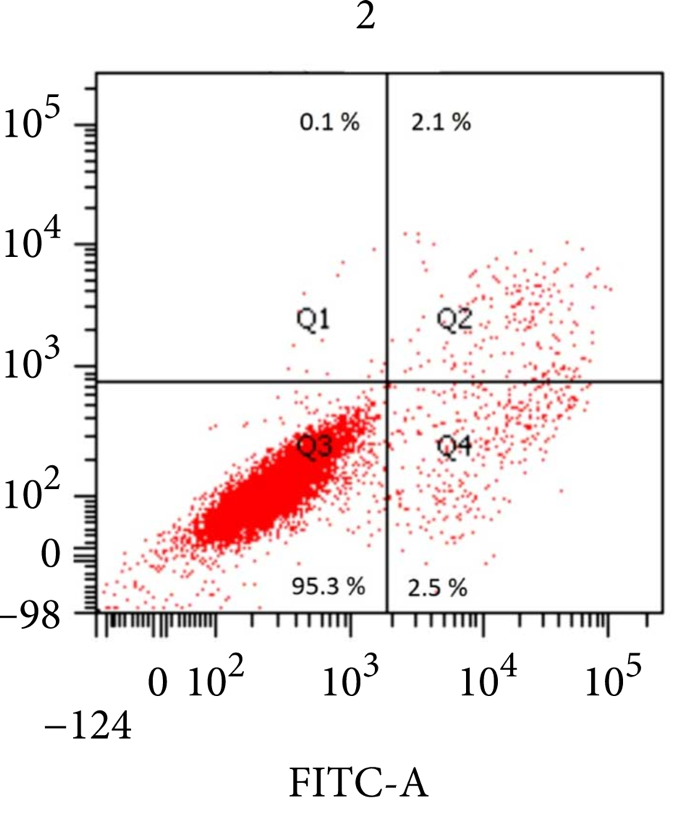
Figure 2A(2) Figure 2B(3)**
