# Supplementary figures and images for: Retracted: Proanthocyanidins Antagonize Arsenic-Induced Oxidative Damage and Promote Arsenic Methylation through Activation of the Nrf2 Signaling Pathway
Source: Oxid Med Cell Longev. 2021 Jan 22;2021:3547620. doi: 10.1155/2021/3547620 (PMC7846395; doi:10.1155/2021/3547620)

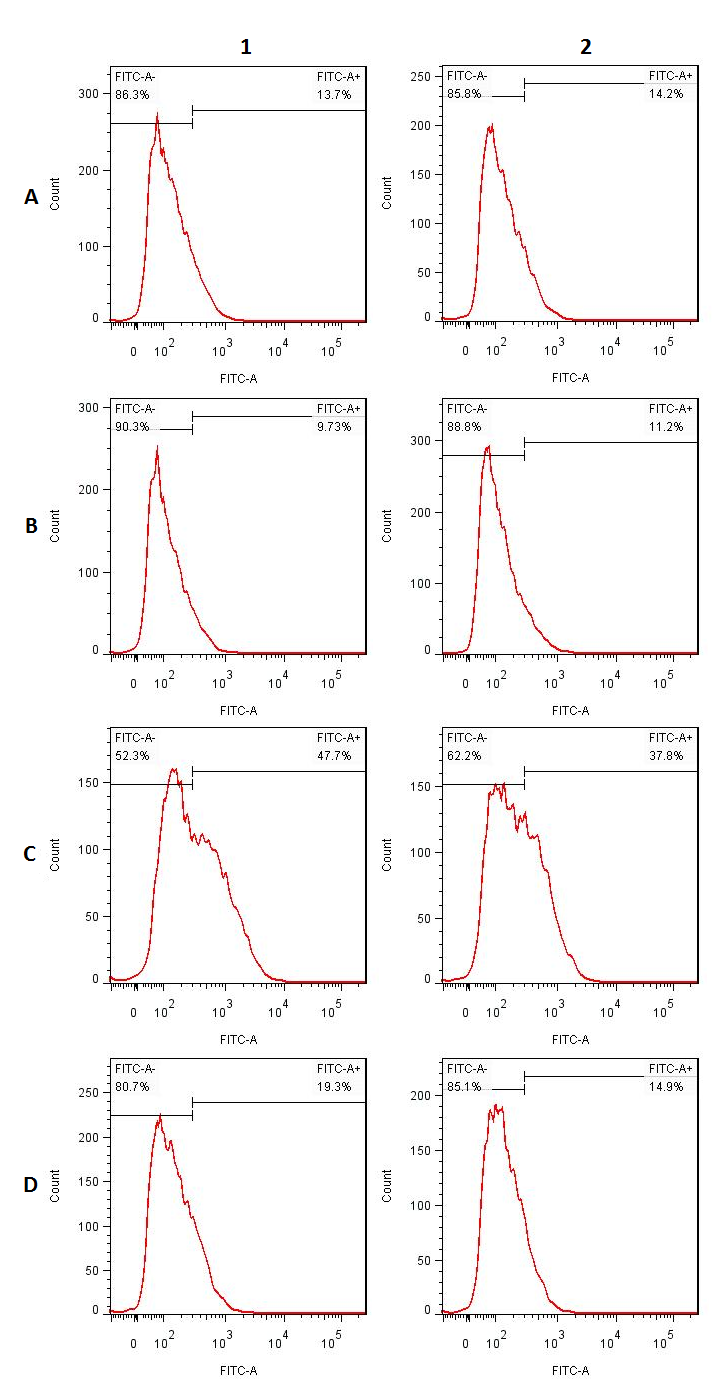

Supplement: Supplementary Materials — Figure duplication in Figure 2 of OMCL/8549035. (Supplementary Materials.docx). Corrected figure files (Supplementary Materials.rar). [file 3547620.f1.zip › 3547620.f1/raw data/2.ROS/flow cytometry(figure4).tif]

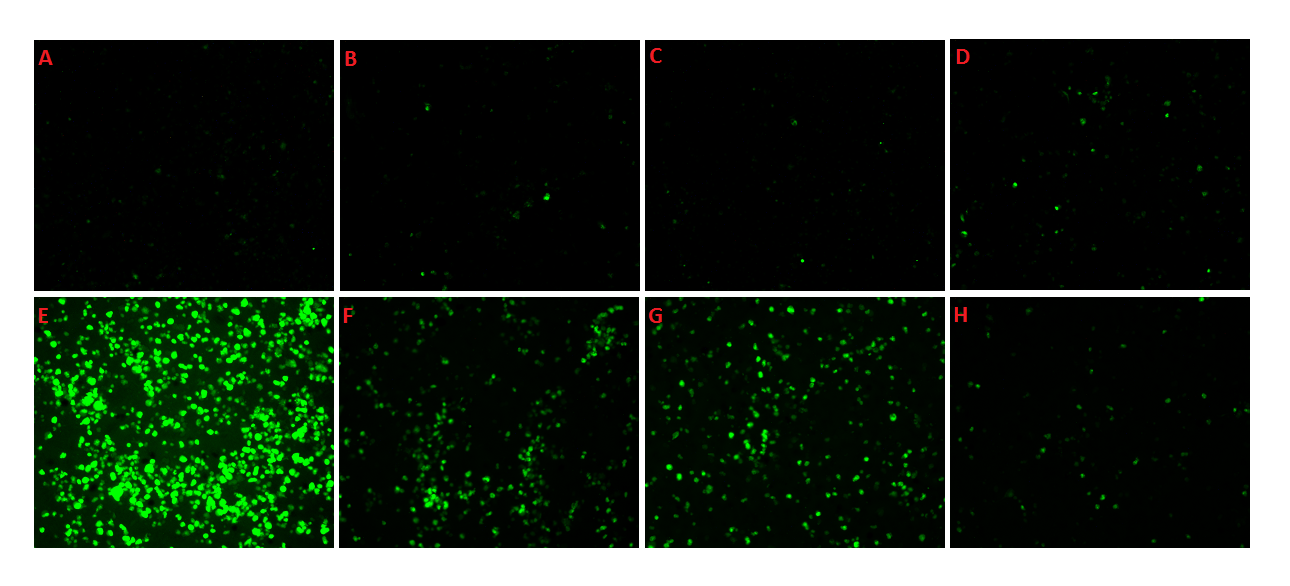

Supplement: Supplementary Materials — Figure duplication in Figure 2 of OMCL/8549035. (Supplementary Materials.docx). Corrected figure files (Supplementary Materials.rar). [file 3547620.f1.zip › 3547620.f1/raw data/2.ROS/fluorescence(figure3).tif]
