# Supplementary material for: Retracted: Proanthocyanidins Antagonize Arsenic-Induced Oxidative Damage and Promote Arsenic Methylation through Activation of the Nrf2 Signaling Pathway
Source: Oxid Med Cell Longev. 2021 Jan 22;2021:3547620. doi: 10.1155/2021/3547620 (PMC7846395; doi:10.1155/2021/3547620)
Supplement: Supplementary Materials — Figure duplication in Figure 2 of OMCL/8549035. (Supplementary Materials.docx). Corrected figure files (Supplementary Materials.rar). [file 3547620.f1.zip › 3547620.f1/raw data/8.The review process/response to reviewers.docx]

October, 16, 2018

Editorial office

Oxidative Medicine and Cellular Longevity

To the Editors:

I would like to submit a revised manuscript for publication in Oxidative Medicine and Cellular Longevity, titled “**Proanthocyanidins antagonises arsenic-induced oxidative damage and promotes arsenic methylation through activation of Nrf2 signalling pathway**”. The paper was co-authored by Mengchuan Xu, Qiang Niu, Yunhua Hu, Gangling Feng, Haixia Wang, Shugang Li. The manuscript number is 8549035.

Thank you very much for your letter, questions and the reviewers’ comments which we found very helpful in improving our manuscript. We have revised the paper according to the comments in a point-by-point format as shown below.

The revised sections and sentences are red colored especially in parts of introduction and discussion because we polish the manuscript.

In terms of English language editing, we have employed an English language service company to edit the whole manuscript.

We thank you and the reviewers for your thoughtful suggestions and insights, which have enriched the manuscript and produced a more balanced and better account of the research. We hope that the revised manuscript is now suitable for publication in your journal.

I look forward to your reply.

Sincerely yours,

Shugang Li, Ph.D.

Department of Public Health

Shihezi University School of Medicine

North 4th Road

Shihezi, Xinjiang China 832003

Phone	: +8618999537041, Fax: +8609932057153

E-mail: lishugang@ymail.com

**Reviewer #1 (No.16):**

The manuscript entitled “Proanthocyanidin antagonises arsenic-induced oxidative damage and promotes arsenic methylation through activation of Nrf2 signalling pathway” reported that GSPE can activate Nrf2 signalling pathway to antagonise As-induced oxidative damage and to promote As methylation metabolism. GSPE may be a potential agent for relieving As-induced hepatotoxicity. The methods used in this manuscript is appropriate. And the conclusions are well supported by the data.

**Response:** Thank you for your careful review of this manuscript. We have made the following modifications one by one to the article according to your suggestions and requirements.

There is a certain novelty and can be published after minor revision.

1.	The authors has reported that Seed Proanthocyanidin Extract Alleviates Arsenic-induced Oxidative Reproductive Toxicity in Male Mice (Biomedical and environmental sciences 2015, 28, 272-280).

In this manuscript, the authors just cited this work as an evidence that arsenic is a poison. I think it is inappropriate. The authors should clarify the relations between this manuscript and the previous work in the introduction section.

**Response:** Thanks for your sugession, your advice is very professional. We replaced the original reference with another one (Chronic Arsenic Poisoning Probably Caused by Arsenic-Based Pesticides: Findings from an Investigation Study of a Household). In addition, we have modified this part according to your suggestion:“Our previous studies have proved that arsenic can caused reproductive toxicity[3, 4], but the mechanism of arsenic toxicity is not completely clear and should be further studied.”(Line37, Page3).

2.	Some errors

Line 548, “We used ML385 was used to inhibit Nrf2 signalling”

**Response:** Thanks for your careful review, we have corrected the mistake. In addition, We have asked a professional language retouching company to modify our manuscript.

**Reviewer #2 (No.18):**

I reviewed the manuscript entitled "Proanthocyanidin antagonises arsenic-induced oxidative damage and promotes arsenic methylation through activation of Nrf2 signalling pathway". Xu et al. examined the effects of grape seed proanthocyanidin extract (GSPE) on oxidative damage and arsenic (As) methylation, and to clarify the role of Nrf2 in the process. The study is well performed, the method is advance and the result reported in the manuscript is interesting and important to the field. The following comments and suggestions are meant to improve the quality of the manuscript:

**Response:** Thank you for your careful review of this manuscript. We have made the following modifications one by one to the article according to your suggestions and requirements.

Introduction

1.The introduction would be more engaging to the reader is some time was spent discussing how proanthocyanidins could be therapeutic, are you saying people should include in your diet? do people know they are being arsenic poisoned, would they take proanthocyanidins to counteract the arsenic? why not just avoid the arsenic? clearly it looks like the proanthocyanidins are therapeutic when taken with arsenic, so how would the model for protection work in a real life situation?

Proanthocyanidins have the anti-oxidant ability, such as can antagoniese zearalenone, lead, aflatoxins. In order to clarify the role of proanthocyanidins, the antioxidant effects of them on these substances should be described in detail.

**Response:** Thanks for your questions and comments. We respond to you one by one as follows.

(1)The introduction would be more engaging to the reader is some time was spent discussing how proanthocyanidins could be therapeutic, are you saying people should include in your diet?

**Response:** Thanks for your professional advice. At present, proanthocyanidins have been widely used in various fields. We have supplemented the Introduction according to your suggestion:“Up till now, there are many health products containing proanthocyanidins in the domestic and foreign markets, such as proanthocyanidins capsule and grape seed antioxidant[11]. Proanthocyanidins can be added to yogurt, cakes and other foods as a food supplement. In addition, proanthocyanidins are widely used in cosmetics, such as hand cream and sunscreen cream[12].”(Line 58, Page3)

(2)do people know they are being arsenic poisoned, would they take proanthocyanidins to counteract the arsenic? why not just avoid the arsenic?

**Response:** Thanks for your quesstions. Endemic arsenic poisoning is a chronic process, and not all people have symptoms of poisoning. At present, many countries in the world still have endemic arseniasis areas, such as India, Bangladesh and China, there are still many people affected by arsenic poisoning. With the improvements in sanitation and medical technology, the concentration of arsenic in drinking water is decreased, and the number of people suffering from arsenic poisoning is reduced. For one reason, however, Endemic arsenic poisoning is caused by arsenic in earth's crust, which is difficult to avoid. For another, due to the lack of water in Xinjiang, high-arsenic groundwater is still used to irrigate farmland, so the arsenic still remains in foods. Therefore, it is necessary to find a new way to treat arsenic poisoning.

(3)clearly it looks like the proanthocyanidins are therapeutic when taken with arsenic, so how would the model for protection work in a real life situation? Proanthocyanidins have the anti-oxidant ability, such as can antagoniese zearalenone, lead, aflatoxins. In order to clarify the role of proanthocyanidins, the antioxidant effects of them on these substances should be described in detail.

**Response:** Thanks for your questions. In the real life situation, proanthocyanidins can be made into drugs or added to foods. People can also eat more proanthocyanidins-rich fruits, like grapes，blueberries and mulberries These measures are helpful to prevent oxidative damage. However, our study only proves that proanthocyanins have some antagonistic effects on arsenic-induced oxidative damage at the cellular level, and there is still a gap between this and the application in human. Therefore, more extensive and in-depth studies should be carried out in the future to clarify the mechanism of proanthocyanidins.

In order to illustrate the antioxidant effect of proanthocyanins, we supplement the preface“Many animal experiments data[13-15] have shown that proanthocyanidins could decrease the content of malondialdehyde (MDA), increase the levels of antioxidant enzymes, such as glutathione peroxidase (GSH-PX), catalase (CAT) and superoxide dismutase (SOD), and alleviate the oxidative damage induced by zearalenone, aflatoxin B1 and lead in mice.”(Line 63, Page3).

Materials and Methods:

1. Why the 25μM arsenic used in your studies? And why choose GSPE (10, 25, and 50 mg/L) for 24 h? why the concentration of ML385 standard was 10 mmol/L, could you give the reader more information?

**Response:** Thanks for your professional questions. We have read many papers before formal experiment, and we found that the concentration of arsenic is 1-50μmol/L. After that, we conducted a preliminary experiment based on the concentration in the literatures, arsenic could cause significant oxidative damage when the concentration is ≥25μmol/L. MTT assay was conducted to test the cell viability. We found that arsenic did not cause massive cell death at 25μmol/L (Figure 1). Therefore, 25μM arsenic was choosed as the final concentration of arsenic. In the same way, we determined the final concentrations and intervention time of GSPE. In addition, ML385 is the standard product purchased from the company (MedChemExpress, Lot No.: 846577-71-9), and the initial concentration is 10mmol/L.

Fig. 1 Viability changes of L-02 cells after arsenic or grape seed procyanidin extract treatment. (Mean & 95% CI). Cell viability was detected by MTT assay. &: 12 h versus control group, P < 0.05; *: 24 h, versus control group, P < 0.05; #: 48 h, versus control group, P < 0.05. CI: Confidence Interval.

2. MTT viability assay methods should described in detail

**Response:** Thanks for your professional sugession. We supplemented the MTT method in detail:“L-02 cell line were seeded (about 5×10^3^ cells were added at 100μL per well) in 96-well plate. Cells were treated with As or GSPE after adherence at 37ºC and 5% CO_2_ atmosphere for 12, 24 and 48 hours. Results were expressed as survival rate, with 100% representing control cells.”(Line115, Page5).

3. Determination of arsenic methylation methods should described in detail

**Response:** Thanks for your professional advice. We supplemented the methods in detail:“Cells and culture media were collected after completion of cell intervention. After cell disruption, the cells (200μL) were filtered with 0.2μm filtration membrane. Chromatographic eluent was 15mmol/L (NH_4_)_2_HPO_4_ solution (pH=6.0, filtered with 0.45μm fiber membrane before use), and the flow rate was 1.0ml /min. The negative pressure of the photomultiplier tube was 285V. The total current of the hollow cathode lamp was 80mA, the auxiliary current was 36mA. The flow rate of carrier gas was 400ml/min. The current-carrying was 7% hydrochloric acid. The reducing agent included 1.5% KBH_4_ solution and 0.35% KOH mixture. The chromatographic column needs to be calibrated for 30 minutes before use.”(Line155, Page7).

4. Please check the method and supplement the test conditions of the instrument.

**Response:** Your advice is really professional. The instruments we used in the experiment mainly include PCR machine, electrophoresis apparatus and high performance liquid chromatograph. PCR instrument and electrophoretic instrument can be tested under regular conditions, in addition, we have supplemented the detection conditions of chromatograph (Line155, Page7).

5. The Nrf2 signaling pathway acts as a classical oxidative stress pathway. The authors should separately test the content of Nrf2 protein in the nucleus and cytoplasm to make the experiment more perfect.

**Response:** Thanks for your professional advice. Nrf2 acts as an antioxidant by entering the nucleus from the cytoplasm. In our study, we tested the total protein and mRNA of Nrf2. Similarly, we found that arsenic intervention reduced the expression of Nrf2, while proanthocyanidins promoted Nrf2 expression. These results showed that the antioxidant effect of proanthocyanidins may be related to Nrf2. Furthermore, ML385 was used to inhibit the expression of Nrf2, it could inhibit Nrf2 at the mRNA level. We found that the antioxidative effect of proanthocyanidins was weaken after Nrf2 inhibition. It further proved that proanthocyanidins could exert its antioxidant effect through Nrf2 pathway. Our experimental results support our conclusions well. In future studies, we will separately test the content of Nrf2 protein in the nucleus and cytoplasm to disclose the mechanism of how proanthocyanidins adjusts the balance of Nrf2 expression in neuclear and cytoplasm.

6. As an inhibitor of Nrf2, ML385 will antagonize arsenic. In order to make the experimental results more accurate, the author should combine the literature or a negative control.

**Response:** Thanks for your suggestion. ML385 is an inhibitor of Nrf2 as report in the literature“Small Molecule Inhibitor of NRF2 Selectively Intervenes Therapeutic Resistance in KEAP1-Deficient NSCLC Tumors”, but there is still a lack of literatures on the relationship between arsenic and ML385. In our study, we found that arsenic could cause oxidative damage, but proanthocyanidins could alleviate the damage induced by arsenic. In order to deeply explore whether proanthocyanidins exerted its antioxidant effect through Nrf2 pathway, we used ML385 to inhibit the expression of Nrf2. Actually, ML385 ascted the similar effect as arsecnic intervention. Our results as follows, showed that both ML385 and/or arsenic intervention could inhibit the expression of Nrf2.

**Fig. 17 mRNA expression of Nrf2 pathway in Nrf2-suppressed cells (mean ± SD, n = 3).** L-02 cells were treated with arsenic and/orgrape seed procyanidin extract (GSPE) for 24 h. *: in Nrf2 normal cells versus control group, *P*< 0.05; #: in Nrf2 normal cells versus arsenic group, *P*< 0.05; a: in Nrf2 inhibition cells versus control group, *P*< 0.05; b: in Nrf2 inhibition cells versus arsenic group, *P*< 0.05. In Nrf2-suppressed cells, GSPE could still mitigate arsenic-induced decrease in mRNA expression of Nrf2 and downstream genes; however, its effect was weaker than that in Nrf2 normal cells.

In the attachment material, we tested the inhibition effect of ML385 on Nrf2 (Figure S1). The optimal intervention concentration (6 μM) and intervention time (72 h) were screened according to Nrf2 expression.

**Figure S1. The inhibition effect of ML385 on Nrf2 (Mean ± SD, n = 3).** *: versus control group of 48 h, *P*< 0.05; #: versus control group of 72 h, *P*< 0.05.ML385 was used to inhibit the expression of Nrf2. The concentration of ML385 standard was 10 mmol/L. The medium without penicillin-streptomycin was used to dilute the standard to 2, 4, 6, 8, and 10 μmol/L. L-02 cells were treated with the diluted standard for 48 and 72 h. The optimal intervention concentration (6 μM) and intervention time (72 h) were screened according to Nrf2 expression.

7. Proanthocyanidin should be Proanthocyanidins, it is not monomers. The Abstract and Discussion sections appear to refer to Proanthocyanidins as a single compound rather than a class of compounds. In contrast, the Introduction refers to proanthocyanidins as a class of compounds. The diversity of the proanthocyanidin class must be clarified.

**Response:** Thanks for your careful review. We ulteriorly refer to the literatures and found that proanthocyanins are a kind of complex flavonoid polymer. Therefore, we revised “proanthocyanidin” to “proanthocyanidins” in our manuscript.

Results:

1. “Fig. 1 (95% CI)”, What is CI?

**Response:** Thanks for your question. CI is the abbreviation of “Confidence Interval”. We have explained it in the caption of Fig. 1.

2. “Fig. 6 Oxidative stress changes in L-02 cells after arsenic and/or grape seed procyanidin extract treatment for 24 h (mean ± SD, n = 3). *: versus control group, P < 0.05; #: versus arsenic group, P < 0.05.”, Display repeat.

**Response:** Thanks for your careful review. We have removed the duplicate caption.

3. Figure 7, what do the different colors represent? And each note of figure should be very clear.

**Response:** Thanks for your question. In Fig. 7, colors don't make any sense. We have removed the colors of Fig. 7.

4. Each figure caption should be detailed for the reader to understand independently of the article.

**Response:** Thanks for your professional advice. We have supplemented the caption of each figure appropriately.

Disscusion

Overall- the discussion lacks a unity of ideas Proanthocyanidins with being a useful agent to minimize toxic arsenic damage---the authors need to relate their findings to potential therapeutic applications otherwise this study has little relevance to reader and a nutritional perspective.

It is recommended that the author supplement the literature to provide more evidence for the test results in this paper.

**Response:** Thanks for your professional advice. We have supplemented the discussion: “At present, endemic arsenism is still a worldwide public health problem, and our study found that proanthocyanins has a good effect on the liver damage caused by arsenic. In addition, proanthocyanins have been widely used in many fields such as medicine, foods and cosmetics, and the safety of proanthocyanins has been well verified[42]. Proanthocyanidins have been used in the adjuvant therapy of patients with obstructive sleep apnea hypopnea syndrome (OSAHS)[43]. Therefore, we believe that proanthocyanins can be used as a potential adjuvant therapy for arsenic poisoning. For example, arsenic poisoning people can eat more fruits riched with proanthocyanins and take some proanthocyanins nourishment while receiving treatment. Procyanidins can also be added to people's daily diet in a right amount.” (Line 592, Page 29).

In addition, we added 7 references into our manuscript. References 11-15 and 42-43.

There are some syntax errors in the manuscript. Authors should carefully read the text and step by sentence to improve the quality of the manuscript.

**Response:** Thanks for your careful review. We have asked a professional language retouching company to modify our manuscript.
